# Supplementary material for: Shell variability in the stem turtles Proterochersis spp
Source: PeerJ. 2018 Dec 21;6:e6134. doi: 10.7717/peerj.6134 (PMC6305121; doi:10.7717/peerj.6134)
Supplement: Supplemental Information 1 — Material, extended methods, supplementary tables, supplementary figures, and supplementary references. [file peerj-06-6134-s001.pdf]

# SHELL VARIABILITY IN THE STEM TURTLES *PROTEROCHERSIS* SPP.

## SUPPLEMENTARY INFORMATION:

## MATERIAL,

## EXTENDED METHODS,

## SUPPLEMENTARY TABLES,

## SUPPLEMENTARY FIGURES,

## AND

## SUPPLEMENTARY REFERENCES

**TOMASZ SZCZYGIELSKI[1,*], JUSTYNA SŁOWIAK[1], AND DAWID DRÓŻDŻ[1]**

[1]Institute of Paleobiology, Polish Academy of Sciences, Twarda 51/55, 00-818 Warsaw, Poland

*E-mail: t.szczygielski@twarda.pan.pl

## MATERIAL

It should be noted that some of the specimens described here (SMNS 17755, SMNS 17756, SMNS 18440, SMNS 50917, SMNS 56606, SMNS 81917, ZPAL V.39/164, ZPAL V.39/197, ZPAL V.39/277, ZPAL V.39/378, ZPAL V.39/381, ZPAL V.39/383, and ZPAL V.39/384), including the ontogenetically youngest individuals, lack any diagnostic characters that would unambiguously support their identification as *Proterochersis robusta* or *P. porebensis*. They all come, however, from Poręba and Murrhardt – the localities which thus far yielded numerous diagnostic specimens referable only to *P. porebensis* and *P. robusta*, respectively. Following Szczygielski and Sulej (in press), we therefore refer them to these taxa. This identification is tentative, but in the light of current knowledge seems to be probable.

### *PROTEROCHERSIS ROBUSTA*

The specimens informative for this study are:

**CSMM uncat.** The holotype of "*Murrhardtia staeschei*" Karl & Tichy, 2000. Found in Mettelberg Quarry, near Murrhardt, Germany. A mostly complete shell (Figs 2A–B, 3A, 9A, S5A, S7A), missing the marginal sections of the carapace (including the cranial edge of the cervical region, but not the last two right marginal scute areas), parts of the first three and of the fifth vertebral scute, pleurals, inframarginals, right bridge and right part of the cranial lobe of the plastron. Figured by Wild (Wild 1987), described and figured by Karl and Tichy (Karl & Tichy 2000) and Szczygielski and Sulej (2016). Carapace approximately 36.5 cm long.

**SMNS 11396.** The holotype of "*Proterochersis intermedia*" Fraas, 1913. Found in Stuttgart-Rohracker, Germany. A fragment of the right side of the plastron (Fig. 3B), with partial areas of the first and second abdominal and femoral, and sutures visible, a natural mold of the visceral surface of the same fragment, a partial natural internal mold of the carapace with impressions of the fourth to 10$^{th}$ dorsal rib, with corresponding rib heads and vertebrae embedded in matrix, and fragments of carapace. The carapace shows no discernible external characters and thus is not informative for this study. Described and figured by Fraas (1913) and Szczygielski and Sulej (2016, in press).

**SMNS 12777.** The holotype of *Proterochersis robusta*. Found in Rudersberg, Germany. A natural mold of the shell, with small fragment of the ninth marginal scute area, complete right side of the pelvis and caudal right side of the plastron preserved (areas of the second abdominal, femoral, anal, intercaudal, base of the caudal, damaged third and fourth inframarginal, fragments of the left anal and caudal scute), and some sutures visible as impressions (Figs 3C, 9B). Figured by Stromer (Stromer 1912) and (in part) by Młynarski (Młynarski 1976). Figured and described in detail by Fraas (1913) and Szczygielski and Sulej (2016, in press).

**SMNS 16442.** Found in Murrhardt, Germany. A damaged cranial part of the carapace (apparently corresponds to the area of the first, and cranial and medial parts of the second vertebral scute, Fig. 2C), an cranial lobe of the plastron (humeral scute area and entoplastron, bearing the medial parts of gular scutes, with epiplastra missing, Fig. 3D), a rock impression of the latter, a caudal lobe of the plastron (area of femorals and anals with a base of the right caudal process, Fig. 3D), a damaged part of the carapace margin with a fragment of the cranial part of the bridge, and some difficult to interpret fragments and impressions in plaster (one of them probably of a marginal sulci), some of these elements with traceable sutures. Due to extensive damage, the fragments of the carapace show little surficial characters. The plastron is better preserved. Described and figured by de Broin (1984) and Szczygielski and Sulej (2016, in press). Histological sections from that specimen were examined by Scheyer and Sander (2007).

**SMNS 16603.** Found in the vicinity of Lorch, Germany. An internal mold of a shell of a young specimen, with a caudal right part of the carapace (Fig. 2D–E), a pelvis, a part of the right axillary buttress and an cranial fragment of the plastron (consisting of the area of the right extragular and gular, a part of the left extragular, and medial parts of humeral scutes, Figs 3E, S7B) preserved. Described and figured by de Broin (1984) and Szczygielski and Sulej (2016, in press).

**SMNS 17561.** Found in Murrhardt, Germany. A virtually complete shell (2F–G, 3F, 9C, S5B, S7C–D, S8A–C), but with substantial parts restored or filled with plaster and paint. The areas of the left extragular, the last pair of inframarginals, right parts of the third and fourth vertebral, caudal part of the second right pleural, right third and fourth pleural, parts of the right supramarginals, right second to fifth and possibly ninth to 12$^{th}$ marginal, left third marginal, and caudal part of the right bridge seem to be at least partially restored. Based on our observations, however, these repairs seem to even out some minor surficial damage suffered by this specimen rather than to fill for any large missing areas of the shell, but their extent is difficult to evaluate. In any case, the morphology is consistent with other specimens of *Proterochersis robusta* and *P. porebensis*, so no major errors were made during that treatment. Carapace approximately 35 cm long. This specimen was described and figured by Gaffney (1986),

Karl and Tichy (Karl & Tichy 2000), and Szczygielski and Sulej (2016). The line drawing of the shell of *Proterochersis robusta* that was first presented in Gaffney (1990), and has frequently been used since, is based mainly on that specimen.

**SMNS 17755.** Found in Murrhardt, Germany. A left bridge and most of the left half of the plastron, consisting of the areas of the sixth to ninth (and part of the 10$^{th}$) marginal, second third, and part of the first supramarginal, second and third inframarginal, fragment of the humeral scute, most of the pectoral, both abdominals, femoral and fragment of anal scute, with visible sutures (Fig. 3G). Large part of this specimen is relatively well-preserved, although the lateral surface is heavily damaged and significant area, especially in cranial section, is restored with plaster. This specimen was described and figured by Szczygielski and Sulej (2016, in press).

**SMNS 17755a.** Found in Murrhardt, Germany. A fragment of carapace, consisting of the areas of the last vertebral scute, a broken off and dislocated fragment of the fourth vertebral scute area, a part of the third and complete fourth left pleural, a fragment of the 10$^{th}$, most of the 11$^{th}$, full 12$^{th}$, and most of the 13$^{th}$ left marginal, with sacrum and dorsal parts of both ilia preserved inside and with visible sutures (Figs 2H, S5C). This specimen was described and figured by Szczygielski and Sulej (in press).

**SMNS 17930.** Found in Oberbrüden, Germany. An internal mold of the carapace with embedded right part of the pelvis and small parts of the right bridge, and a large part of the carapace (Figs 2I–J, 6, S8D), including the areas of the cervical scute, complete first vertebral and increasingly more fragmentary second, third, fourth, and small fragment of the fifth vertebral scute, complete first pair of pleurals, fragmentary second, third, and fourth right pleural, damaged right supramarginals, and marginal scutes: the first and the second, a fragment of the third, complete fourth, fragmentary fifth and 10$^{th}$, complete 11$^{th}$, and a fragment of the 12$^{th}$. Part of the mid-section and fragments near the right cranial and caudal margin are restored with plaster. Carapace approximately 36 cm long.

**SMNS 18440.** Found in Murrhardt, Germany. A left bridge fragment, comprising the area of small caudal fragments of the pectoral scute, nearly complete first and complete second abdominal scute, most of the femoral scute, a part of the first and complete second, third, and (poorly preserved) fourth inframarginal, a fragment of the fifth and 10$^{th}$ marginal scute, and complete sixth, seventh, eighth, and ninth marginals, complete three supramarginals, and fragments of the second and third pleural scute, with sutures visible (Figs 2K, 3H). Apart from several breaks, it is well-preserved. This specimen was described and figured by Szczygielski and Sulej (in press).

**SMNS 50917.** Found in Murrhardt, Germany. A fragmentary plastron consisting of an area of complete left femoral scute and fragments of the left pectoral, the first and the second left abdominal, the right femoral and right and left anal scutes (Fig. 3I). Viscerally, a base of the pelvis and the epipubic process are preserved. Associated is an unspecified, poorly-preserved shell fragment, possibly from the bridge area.

**SMNS 56606.** Found in Murrhardt, Germany. A mostly complete pelvis of a young individual with attached badly damaged caudal part of the carapace (natural surface preserved only in the partial area of the last and the second-to-last left marginal scute and in some places along the rim of the caudal notch), at least one sacral vertebra and rib pair, caudal segment of the dorsal vertebral column (approximately two complete and caudal half of the third centrum with associated rib heads), and poorly preserved caudal part of the plastron, consisting of the areas of mostly intact intercaudal scute and damaged caudal sections of the anal scutes, with caudal processes broken off (Fig. 3J).

**SMNS 81917.** Found in Murrhardt, Germany. A partially preserved plastral bone (hypoplastron or hypoplastron) of a juvenile exposed in visceral view (Fig. S6A).

### *PROTEROCHERSIS POREBENSIS*

All of the existing specimens (ZPAL V.39/1–28, ZPAL V.39/34, ZPAL V.39/48–72, ZPAL V.39/155–300, ZPAL V.39/331–366, ZPAL V.39/370, ZPAL V.39/373–404, ZPAL V.39/416–420, and uncatalogued) were studied. The specimens informative for this study are:

**ZPAL V.39/1.** A fragment of a middle right section of a carapace consisting of partial areas of the first and the second vertebral scute, including proximal parts of three ribs (Fig. S4G–H).

**ZPAL V.39/2.** A fragment of a middle left section of a carapace, consisting of partial areas of the second and the third vertebral scute, including proximal parts of five ribs (Fig. S4A–B).

**ZPAL V.39/3.** A large part of an isolated costal with sutural edges. This specimen was described and figured by Szczygielski and Sulej (in press).

**ZPAL V.39/4.** An isolated neural bone with transverse, ω-shaped sulcus. This specimen was described and figured by Szczygielski and Sulej (in press).

**ZPAL V.39/6.** Caudal right section of a carapace, including partial area of the fifth vertebral scute, two last marginal scute areas, and partial area of the third-to-last marginal scute, with part of the caudal process of ilium (Figs S5D, S8E–F).

**ZPAL V.39/8.** A fragment of a left bridge region, consisting of the areas of complete first and cranial part of the second supramarginal scute, a part of the fifth, complete sixth, and part of the seventh marginal scute, a fragment of the first pleural scute, and minute fragments of the axillary and the first two inframarginal scutes. This specimen was described and figured by Szczygielski and Sulej (in press).

**ZPAL V.39/18.** Caudal right part of the carapace, consisting of the last marginal scute area, partial area of the second-to-last and third-to-last marginal scute, partial fourth and fifth vertebral scute areas, and partial area of the fourth right pleural, with part of the caudal process of ilium (Fig. S5E).

**ZPAL V.39/22.** A fragment of the cranial left nuchal part of the carapace, including left half of the cervical scute, part of the first vertebral scute, and parts of the cranial two marginals (Fig. S3A). This specimen was described and figured by Szczygielski and Sulej (in press).

**ZPAL V.39/23.** A fragment of the caudal left carapacial rim, consisting of the area of the last marginal, a caudal part of the preceding marginal, and a small fragment of the last vertebral scute, with sutural edge (Fig. S5F). This specimen was described and figured by Szczygielski and Sulej (in press).

**ZPAL V.39/34.** A nearly complete shell of a juvenile (Figs 4A–B, 5A, 9K, S4C, S5G, S7E–G). Carapace approximately 28 cm long. This specimen was described and figured by Sulej et al. (2012) and Szczygielski and Sulej (2016).

**ZPAL V.39/48.** The holotype of *Proterochersis porebensis*. A nearly complete shell (Figs 4C–D, 5B, 9G, S2A–B, S5H, S7H–J), pelvis, left scapulocoracoid, and right femur. Carapace approximately 42.5 cm long. This specimen was described and figured by Szczygielski and Sulej (2016) and Szczygielski (2017).

**ZPAL V.39/49.** A mostly complete shell (Figs 4E–F, 5C, 9D, S2C–F, S5I, S7K–M) and pelvis. Carapace approximately 49 cm long. This specimen was described and figured by Szczygielski and Sulej (2016) and Szczygielski (2017).

**ZPAL V.39/56.** Left caudal process of plastron (Fig. 9L–N).

**ZPAL V.39/57.** Cranial left part of the carapace, consisting of partial areas of the cervical scute, first vertebral, first left pleural, and marginal scute areas: complete first, fragmentary second, complete third and fourth, and partial fifth (Figs S1N, S3B).

**ZPAL V.39/59.** Caudal left part of the carapace, consisting of two last marginal scute areas and partial last vertebral scute area, with part of the caudal process of ilium (Fig. S5J). This specimen was described and figured by Szczygielski and Sulej (in press).

**ZPAL V.39/60.** A fragment of a carapace, consisting of a complete ninth scute area and fragmentary areas of the third supramarginal scute and the third pleural scute (Fig. S1O–P).

**ZPAL V.39/63.** Caudal right part of the carapace, consisting of partial areas of the fourth and fifth vertebral scutes, and a posterodorsal part of the fourth pleural scute area, with attached dorsal section of the right ilium (Fig. S1A–B).

**ZPAL V.39/66.** Caudal plastral lobe consisting of the area of the caudal part of the anal scutes, nearly complete intercaudal scute, proximal part of the right and complete left caudal scute, with the basal part of the ischia attached to the visceral surface (Fig. 9J).

**ZPAL V.39/68.** Caudal plastral lobe consisting of the area of the caudal part of the anal scutes, the complete intercaudal scute, and proximal parts of caudal scutes, with the basal part of the ischia attached to the visceral surface (Fig. 9H). Although the caudal scutes are broken at the base, it is clear that they were elongated and aligned caudally. This specimen was described and figured by Szczygielski and Sulej (in press).

**ZPAL V.39/69.** Caudal plastral lobe consisting of the area of the anal, intercaudal, and caudal scutes, with bases of the lateral pubic processes and basal part of the ischia and pubis attached to the visceral surface (Fig. 9F). This specimen was described and figured by Szczygielski and Sulej (in press).

**ZPAL V.39/70.** Caudal plastral lobe consisting of the area of the caudal part of the anal scutes, nearly complete intercaudal scute, proximal part of the left and complete right caudal scute, with the basal part of the ischia attached to the visceral surface (Fig. 9I).

**ZPAL V.39/71.** Caudal plastral lobe consisting of the area of the caudal part of the anal scutes, nearly complete intercaudal scute, proximal part of the left and complete right caudal scute, with the basal part of the ischia attached to the visceral surface (Fig. 9E).

**ZPAL V.39/72.** A mostly complete carapace, missing only parts of the areas of the third, fourth, and fifth vertebral scute, fragments of the second left and third and fourth right pleural scute, part of the second and most of the third right supramarginal scute, as well as the eighth and cranial part of the ninth right marginal scute (Figs 4G–H, S4D, S5K). Associated with a proximal caudal vertebra. Carapace approximately 44.5 cm long. This specimen was described and figured in Szczygielski and Sulej (2016) and Szczygielski (2017).

**ZPAL V.39/157.** A fragmentary plastron, consisting of partial sections of the right femoral and right anal scute areas with attached right lateral pubic process.

**ZPAL V.39/165.** A partially preserved plastral bone (likely right hyoplastron) of a juvenile.

**ZPAL V.39/169.** A fragment of the dorsal vertebral column consisting of one complete and small fragments of two surrounding vertebrae and an attached part of a carapace with intervertebral sulcus (Figs S2G, S4E–F).

**ZPAL V.39/176.** A fragment of a costal with sutural edges (Fig. S1E).

**ZPAL V.39/186.** Left gular tubercle of a large specimen (Fig. S7N–Q). This specimen was described and figured by Szczygielski and Sulej (in press).

**ZPAL V.39/187.** A fragment of the cranial plastral lobe consisting of the area of the right gular and extragular (Fig. S7R–T).

**ZPAL V.39/189.** A fragment of the cranial plastral lobe consisting of the area of the right gular and medial part of the right extragular (Fig. S7U–X).

**ZPAL V.39/197.** A partially preserved plastral bone (likely right hyoplastron) of a juvenile (Fig. S6C).

**ZPAL V.39/199.** Left caudal process of plastron (Fig. 9O–Q).

**ZPAL V.39/200.** (?)Left caudal process of plastron (Fig. 9R–T).

**ZPAL V.39/213.** A fragment of the caudal left carapacial rim, consisting of the area of the last marginal, a caudal edge of the preceding marginal, and a small fragment of the last vertebral scute, with suture running at the base of the peripheral (Fig. S5L).

**ZPAL V.39/277.** A partially preserved plastral bone of a juvenile (Fig. S6B).

**ZPAL V.39/333.** A fragment of the right part of the cranial plastral lobe, consisting of the complete right gular, extragular, and an cranial part of the right humeral scute area, and small fragment of the left humeral scute area (Fig. S7Y–A′).

**ZPAL V.39/377.** An unankylosed fragment of the dorsal vertebral column of a young specimen, consisting of one complete vertebra and an cranial half of a succeeding vertebra (Figs S1H–K, S2I).

**ZPAL V.39/378.** An ankylosed fragment of the dorsal vertebral column, consisting of one complete vertebra and fragments of a preceding and succeeding vertebra with associated rib heads (Figs S1F–G, S2J).

**ZPAL V.39/379.** A fragment of the right part of the cranial plastral lobe, consisting of the complete right gular, extragular, and an cranial part of the right humeral scute area (Fig. S7B′–D′).

**ZPAL V.39/380.** Caudal right part of the carapace, consisting of two last marginal scute areas and partial last vertebral scute area, with part of the caudal process of ilium (Fig. S5M).

**ZPAL V.39/381.** A fragmentary costal of a hatchling (Fig. S1C–D).

**ZPAL V.39/382.** A fragment of a large costal.

**ZPAL V.39/383.** A partially preserved plastral bone of a juvenile.

**ZPAL V.39/384.** A partially preserved plastral bone of a juvenile (Fig. S6D).

**ZPAL V.39/385.** A nearly complete cranial plastral lobe with bases of the dorsal processes of the epiplastra and complete caudal process of the entoplastron preserved on the visceral side, and areas of both gular, both extragular, two supernumerary, right humeral and right pectoral scutes, as well as partially preserved left humeral and pectoral, and the first pair of abdominals (Figs 7, S7E′–G′).

**ZPAL V.39/386.** Caudal left part of the carapace, consisting of partial area of the last and third-to-last marginal scute, complete area of the second to last marginal scute, and partial fourth and fifth vertebral scute areas, with part of the caudal process of ilium (Fig. S5N).

**ZPAL V.39/387.** A fragment of the cranial plastral lobe consisting of the area of the left gular and extragular, and an cranial part of the left humeral scute area (Fig. S7H′–J′).

**ZPAL V.39/388.** A fragment of the cranial plastral lobe consisting of the area of the right gular and extragular (Fig. S7K′–M′).

**ZPAL V.39/390.** Cranial left part of carapace consisting of partial areas of the cervical scute, the first vertebral scute, and damaged two cranialmost left marginal scutes (Fig. S1L–M).

**ZPAL V.39/402.** Caudal part of the carapace consisting of the middle area of the fifth vertebral with coosified last dorsal vertebra and both sacral vertebrae (Fig. S2K–L).

**ZPAL V.39/420.** A fragment of the cranial plastral lobe consisting of the area of the left gular and extragular (Fig. S7N′–P′)

## TABLES

**Table S1.** *Proterochersis robusta*, carapace scutes. **+** – preserved whole or nearly whole; **P** – partially preserved; ***** – preserved only as an impression or damaged bone but providing at least partial data about the outline; **-** – not preserved or insignificant part preserved. X indicates lack of the fifteenth marginal. **R** means right, **L** means left side of the body. Note that the numbering of caudalmost marginals is uncertain if the twelfth one is not preserved at least in most part. SMNS 17561 has most of its scutes restored – these restorations seem to be mostly superficial and close to the original shape of the elements, but their extent is difficult to evaluate.

| Specimen | Side | Cervical | Vertebral | | | | | Pleural | | | | Supramarginal | | | Marginal | | | | | | | | | | | | | | |
|---|---|---|---|---|---|---|---|---|---|---|---|---|---|---|---|---|---|---|---|---|---|---|---|---|---|---|---|---|---|
| | | | 1 | 2 | 3 | 4 | 5 | 1 | 2 | 3 | 4 | 1 | 2 | 3 | 1 | 2 | 3 | 4 | 5 | 6 | 7 | 8 | 9 | 10 | 11 | 12 | 13 | 14 | 15 |
| CSMM | R | P | P | P | P | + | + | - | - | - | P | - | - | - | - | - | - | - | - | - | - | - | - | - | - | - | - | - | ? |
| | L | P | + | P | P | + | P | - | - | - | - | - | - | - | - | P | P | - | - | - | - | - | - | - | - | ?P | ?+ | ?+ | ?X |
| SMNS 11396 | R | - | - | - | P | P | - | - | - | - | - | - | - | - | - | - | - | - | - | - | - | - | - | - | - | - | - | - | ? |
| | L | - | - | - | P | - | - | - | - | - | - | - | - | - | - | - | - | - | - | - | - | - | - | - | - | - | - | - | ? |
| SMNS 16442 | R | - | P | P | P | - | - | - | - | - | - | - | - | - | - | - | - | - | - | - | - | - | - | - | - | - | - | - | ? |
| | L | - | P | P | - | - | - | - | - | - | - | - | - | - | - | - | - | - | - | - | - | - | - | - | - | - | - | - | ? |
| SMNS 16603 | R | - | - | - | - | P | P | - | - | P | P | - | - | - | - | - | - | - | - | - | - | - | - | - | - | - | - | - | ? |
| | L | - | - | - | - | - | - | - | - | - | - | - | - | - | - | - | - | - | - | - | - | - | - | - | - | - | - | - | ? |
| SMNS 17561 | R | + | + | + | + | + | + | + | + | + | + | + | + | + | P | + | P | + | + | + | + | + | + | + | + | P | P | + | X |
| | L | + | + | + | + | + | + | + | + | + | + | + | + | + | + | + | + | P | P | + | + | + | P | P | + | P | P | P | X |
| SMNS 17755 | R | - | - | - | - | - | - | - | - | - | - | - | - | - | - | - | - | - | - | - | - | - | - | - | - | - | - | - | ? |
| | L | - | - | - | - | - | - | - | - | - | - | P | P | P | - | - | - | - | * | P | P | P | P | P | - | - | - | - | ? |
| SMNS 17755a | R | - | - | - | - | P | P | - | - | - | - | - | - | - | - | - | - | - | - | - | - | - | - | - | - | - | - | - | ? |
| | L | - | - | - | - | - | P | - | - | P | + | - | - | - | - | - | - | - | - | - | - | - | - | P | P | + | + | - | ? |
| SMNS 17930 | R | + | P | P | P | P | P | P | + | P | P | P | P | P | + | + | P | + | P | - | - | - | - | P | + | P | - | - | ? |
| | L | P | + | P | P | - | - | - | - | - | - | - | - | - | P | - | - | - | - | - | - | - | - | - | - | - | - | - | ? |
| SMNS 18440 | R | - | - | - | - | - | - | - | - | - | - | - | - | - | - | - | - | - | - | - | - | - | - | - | - | - | - | - | ? |
| | L | - | - | - | - | - | - | - | P | P | - | P | + | P | - | - | - | - | P | + | + | + | P | P | - | - | - | - | ? |
| SMNS 56606 | R | - | - | - | - | - | * | - | - | - | - | - | - | - | - | - | - | - | - | - | - | - | - | - | - | - | - | - | ? |
| | L | - | - | - | - | - | * | - | - | - | - | - | - | - | - | - | - | - | - | - | - | - | - | - | - | - | - | - | ? |

**Table S2.** *Proterochersis robusta*, plastron scutes. **+** – preserved whole or nearly whole; **P** – partially preserved; ***** – preserved only as an impression or damaged bone but providing at least partial data about the outline; **-** – not preserved or insignificant part preserved. **R** means right, **L** means left side of the body. SMNS 17561 has most of its scutes restored – these restorations seem to be mostly superficial and close to the original shape of the elements, but their extent is difficult to evaluate.

| Specimen | Side | Extragular | Gular | Humeral | Pectoral | Abdominal | | Femoral | Anal | Caudal | Intercaudal | Axillary | Inframarginal | | | |
|---|---|---|---|---|---|---|---|---|---|---|---|---|---|---|---|---|
| | | | | | | 1 | 2 | | | | | | 1 | 2 | 3 | 4 |
| CSMM | R | - | P | P | P | P | P | + | + | + | + | - | - | - | - | - |
| | L | P | + | + | + | + | + | + | + | + | | + | - | P | - | - |
| SMNS 11396 | R | - | - | - | - | P | P | P | - | - | - | - | - | - | - | - |
| | L | - | - | - | - | - | - | - | - | - | | - | - | - | - | - |
| SMNS 12777 | R | - | - | - | - | - | + | + | + | P | + | - | - | - | - | * |
| | L | - | - | - | - | - | - | - | * | * | | - | - | - | - | - |
| SMNS 16442 | R | - | P | P* | * | - | - | P | + | - | - | - | - | - | - | - |
| | L | - | P | P* | * | - | - | P | + | P | | - | - | - | - | - |
| SMNS 16603 | R | + | P | P | - | - | - | - | - | - | - | P | - | - | - | - |
| | L | - | - | P | - | - | - | - | - | - | | - | - | - | - | - |
| SMNS 17561 | R | P | + | + | + | + | P | + | + | + | + | + | + | + | + | * |
| | L | + | + | + | + | + | + | + | + | + | | + | + | + | + | * |
| SMNS 17755 | R | - | - | - | - | - | - | - | - | - | - | - | - | - | + | + |
| | L | - | - | P | P | P | + | + | P | - | | - | - | - | - | - |
| SMNS 18440 | R | - | - | - | - | - | - | - | - | - | - | - | - | - | - | - |
| | L | - | - | - | - | P | + | P | - | - | | - | P | + | + | + |
| SMNS 50917 | R | - | - | - | - | - | - | - | P | - | - | - | - | - | - | - |
| | L | - | - | - | - | P | P | + | P | - | | - | - | - | - | - |
| SMNS 56606 | R | - | - | - | - | - | - | - | P | - | + | - | - | - | - | - |
| | L | - | - | - | - | - | - | - | P | P | | - | - | - | - | - |

**Table S3.** *Proterochersis porebensis*, carapace scutes. **+** – preserved whole or nearly whole; **P** – partially preserved; ***** – preserved only as an impression or damaged bone but providing at least partial data about the outline; **-** – not preserved or insignificant part preserved. X indicates lack of the fifteenth marginal. **R** means right, **L** means left side of the body. Note that the numbering of caudalmost marginals is uncertain if the twelfth one is not preserved at least in most part. See text for discussion on ZPAL V.39/34 marginal scutes.

| Specimen | Side | Cervical | Vertebral | | | | | Pleural | | | | Supramarginal | | | Marginal | | | | | | | | | | | | | | |
|---|---|---|---|---|---|---|---|---|---|---|---|---|---|---|---|---|---|---|---|---|---|---|---|---|---|---|---|---|---|
| | | | 1 | 2 | 3 | 4 | 5 | 1 | 2 | 3 | 4 | 1 | 2 | 3 | 1 | 2 | 3 | 4 | 5 | 6 | 7 | 8 | 9 | 10 | 11 | 12 | 13 | 14 | 15 |
| ZPAL V.39/1 | R | - | P | P | - | - | - | - | - | - | - | - | - | - | - | - | - | - | - | - | - | - | - | - | - | - | - | - | ? |
| | L | - | - | - | - | - | - | - | - | - | - | - | - | - | - | - | - | - | - | - | - | - | - | - | - | - | - | - | ? |
| ZPAL V.39/2 | R | - | - | - | - | - | - | - | - | - | - | - | - | - | - | - | - | - | - | - | - | - | - | - | - | - | - | - | ? |
| | L | - | - | P | P | P | - | - | - | - | - | - | - | - | - | - | - | - | - | - | - | - | - | - | - | - | - | - | ? |
| ZPAL V.39/5 | R | - | - | - | - | - | - | - | - | - | - | - | - | - | - | - | - | - | - | - | - | - | - | - | - | - | - | - | ? |
| | L | - | - | - | - | - | - | - | - | - | - | - | - | P | - | - | - | - | - | - | - | + | P | - | - | - | - | - | ? |
| ZPAL V.39/6 | R | - | - | - | - | - | P | - | - | - | - | - | - | - | - | - | - | - | - | - | - | - | - | - | - | ?P | ?+ | ?+ | ?X |
| | L | - | - | - | - | - | - | - | - | - | - | - | - | - | - | - | - | - | - | - | - | - | - | - | - | - | - | - | ? |
| ZPAL V.39/8 | R | - | - | - | - | - | - | - | - | - | - | - | - | - | - | - | - | - | - | - | - | - | - | - | - | - | - | - | ? |
| | L | - | - | - | - | - | - | - | - | - | - | + | P | - | - | - | - | - | P | + | P | - | - | - | - | - | - | - | ? |
| ZPAL V.39/18 | R | - | - | - | - | P | P | - | - | - | P | - | - | - | - | - | - | - | - | - | - | - | - | - | - | P | P | + | X |
| | L | - | - | - | - | - | - | - | - | - | - | - | - | - | - | - | - | - | - | - | - | - | - | - | - | - | - | - | ? |
| ZPAL V.39/21 | R | - | - | - | - | - | - | - | - | - | - | - | - | - | - | - | - | - | P | P | - | - | - | - | - | - | - | - | ? |
| | L | - | - | - | - | - | - | - | - | - | - | - | - | - | - | - | - | - | - | - | - | - | - | - | - | - | - | - | ? |
| ZPAL V.39/22 | R | - | - | - | - | - | - | - | - | - | - | - | - | - | - | - | - | - | - | - | - | - | - | - | - | - | - | - | ? |
| | L | + | P | - | - | - | - | - | - | - | - | - | - | - | P | P | - | - | - | - | - | - | - | - | - | - | - | - | ? |
| ZPAL V.39/23 | R | - | - | - | - | - | - | - | - | - | - | - | - | - | - | - | - | - | - | - | - | - | - | - | - | - | - | - | ? |
| | L | - | - | - | - | - | P | - | - | - | - | - | - | - | - | - | - | - | - | - | - | - | - | - | - | - | ?P | ?+ | ?X |
| ZPAL V.39/34 | R | + | + | P | P | P | P | P | P | P | P | * | P | + | ? | + | P | P | P | P | + | + | + | + | + | P | + | P | X |
| | L | + | + | P | P | P | P | P | P | P | + | * | + | + | ? | + | P | P | P | P | P | P | + | P | + | P | + | + | X |
| ZPAL V.39/48 | R | + | + | P | + | + | + | + | + | + | + | + | + | + | P | P | P | P | P | P | + | + | + | + | P | P | P | + | + |
| | L | + | + | + | + | + | + | + | + | + | + | P | + | + | P | + | P | P | P | + | + | + | + | P | P | P | P | + | + |
| ZPAL V.39/49 | R | + | + | + | + | P | P | + | + | + | P | + | + | + | + | + | P | + | P | P | + | + | + | + | P | P | P | P | X |
| | L | + | + | P | P | P | P | + | P | P | P | + | + | P | P | + | P | P | + | + | P | P | + | + | + | P | + | + | X |
| ZPAL V.39/54 | R | - | - | - | - | - | - | - | - | - | - | - | - | - | - | - | - | - | - | - | - | - | - | - | - | - | - | - | ? |
| | L | - | - | - | - | - | - | - | - | - | - | - | - | - | - | - | - | - | - | - | - | - | - | - | - | - | - | ?+ | ?X |

| Specimen | Side | Cervical | Vertebral | | | | | Pleural | | | | Supramarginal | | | Marginal | | | | | | | | | | | | | | |
|---|---|---|---|---|---|---|---|---|---|---|---|---|---|---|---|---|---|---|---|---|---|---|---|---|---|---|---|---|---|
| | | | 1 | 2 | 3 | 4 | 5 | 1 | 2 | 3 | 4 | 1 | 2 | 3 | 1 | 2 | 3 | 4 | 5 | 6 | 7 | 8 | 9 | 10 | 11 | 12 | 13 | 14 | 15 |
| ZPAL V.39/57 | R | - | - | - | - | - | - | - | - | - | - | - | - | - | - | - | - | - | - | - | - | - | - | - | - | - | - | - | ? |
| | L | P | P | - | - | - | - | P | - | - | - | - | - | - | + | P | + | + | P | - | - | - | - | - | - | - | - | - | ? |
| ZPAL V.39/59 | R | - | - | - | - | - | - | - | - | - | - | - | - | - | - | - | - | - | - | - | - | - | - | - | - | - | - | - | ? |
| | L | - | - | - | - | - | P | - | - | - | - | - | - | - | - | - | - | - | - | - | - | - | - | - | - | - | ?P | ?+ | ?X |
| ZPAL V.39/60 | R | - | - | - | - | - | - | - | - | - | - | - | - | - | - | - | - | - | - | - | - | - | - | - | - | - | - | - | ? |
| | L | - | - | - | - | - | - | - | - | P | - | - | - | P | - | - | - | - | - | - | - | - | + | - | - | - | - | - | ? |
| ZPAL V.39/63 | R | - | - | - | - | P | P | - | - | - | P | - | - | - | - | - | - | - | - | - | - | - | - | - | - | - | - | - | ? |
| | L | - | - | - | - | - | - | - | - | - | - | - | - | - | - | - | - | - | - | - | - | - | - | - | - | - | - | - | ? |
| ZPAL V.39/67 | R | - | - | - | - | - | - | - | - | - | - | - | - | - | - | - | - | - | - | - | - | - | - | - | - | - | - | - | ? |
| | L | - | - | - | - | - | - | - | - | P | - | - | - | P | - | - | - | - | - | - | - | - | + | - | - | - | - | - | ? |
| ZPAL V.39/72 | R | P | + | + | P | P | P | + | + | P | P | + | + | P | + | + | + | + | + | P | P | - | P | P | P | P | + | + | X |
| | L | P | + | + | P | P | P | + | P | + | + | + | P | + | P | P | + | P | P | P | + | P | + | + | P | P | + | + | X |
| ZPAL V.39/158 | R | - | - | - | - | - | - | - | - | - | - | - | - | - | - | - | - | - | - | - | - | - | - | - | - | - | - | - | ? |
| | L | - | P | - | - | - | - | P | - | - | - | - | - | - | - | - | P | + | P | - | - | - | - | - | - | - | - | - | ? |
| ZPAL V.39/160 | R | - | - | - | - | - | - | - | P | - | - | P | + | P | - | - | - | - | - | P | + | P | - | - | - | - | - | - | ? |
| | L | - | - | - | - | - | - | - | - | - | - | - | - | - | - | - | - | - | - | - | - | - | - | - | - | - | - | - | ? |
| ZPAL V.39/161 | R | - | - | - | - | - | - | - | - | - | - | - | - | - | - | - | - | - | - | - | - | - | - | - | - | - | - | - | ? |
| | L | + | P | - | - | - | - | - | - | - | - | - | - | - | P | - | - | - | - | - | - | - | - | - | - | - | - | - | ? |
| ZPAL V.39/167 | R | - | - | - | - | - | - | - | - | - | - | - | - | - | - | - | - | - | - | - | - | - | - | - | - | - | - | - | ? |
| | L | - | - | - | - | - | - | - | - | - | P | - | - | - | - | - | - | - | - | - | - | - | - | P | P | - | - | - | ? |
| ZPAL V.39/168 | R | - | - | - | - | - | - | - | - | - | - | - | - | - | - | - | - | - | - | - | - | - | - | - | - | - | - | - | ? |
| | L | - | - | - | - | - | - | - | - | - | - | - | P | - | - | - | - | - | - | P | P | - | - | - | - | - | - | - | ? |
| ZPAL V.39/173 | R | - | - | - | - | - | - | - | - | - | - | - | - | - | - | - | - | - | - | - | - | - | - | - | - | - | - | - | ? |
| | L | - | - | - | - | - | - | - | - | - | - | - | - | - | - | - | - | - | - | - | - | P | P | - | - | - | - | - | ? |
| ZPAL V.39/213 | R | - | - | - | - | - | - | - | - | - | - | - | - | - | - | - | - | - | - | - | - | - | - | - | - | - | - | - | ? |
| | L | - | - | - | - | - | P | - | - | - | - | - | - | - | - | - | - | - | - | - | - | - | - | - | - | - | - | ?+ | ?X |
| ZPAL V.39/370 | R | - | - | - | - | - | - | - | - | - | - | - | - | - | - | - | - | - | - | - | - | - | - | - | - | - | - | - | ? |
| | L | - | - | - | - | - | P | - | - | - | - | - | - | - | - | - | - | - | - | - | - | - | - | - | - | - | - | - | ? |
| ZPAL V.39/380 | R | - | - | - | - | - | P | - | - | - | - | - | - | - | - | - | - | - | - | - | - | - | - | - | - | - | ?+ | ?+ | ?X |
| | L | - | - | - | - | - | - | - | - | - | - | - | - | - | - | - | - | - | - | - | - | - | - | - | - | - | - | - | ? |
| ZPAL V.39/386 | R | - | - | - | - | - | - | - | - | - | - | - | - | - | - | - | - | - | - | - | - | - | - | - | - | - | - | - | ? |
| | L | - | - | - | - | - | P | - | - | - | - | - | - | - | - | - | - | - | - | - | - | - | - | - | - | P | + | P | X |

| Specimen | Side | Cervical | Vertebral | | | | | Pleural | | | | Supramarginal | | | Marginal | | | | | | | | | | | | | | |
|---|---|---|---|---|---|---|---|---|---|---|---|---|---|---|---|---|---|---|---|---|---|---|---|---|---|---|---|---|---|
| | | | 1 | 2 | 3 | 4 | 5 | 1 | 2 | 3 | 4 | 1 | 2 | 3 | 1 | 2 | 3 | 4 | 5 | 6 | 7 | 8 | 9 | 10 | 11 | 12 | 13 | 14 | 15 |
| ZPAL V.39/390 | R | P | - | - | - | - | - | - | - | - | - | - | - | - | - | - | - | - | - | - | - | - | - | - | - | - | - | - | ? |
| | L | + | P | - | - | - | - | - | - | - | - | - | - | - | - | - | - | - | - | - | - | - | - | - | - | - | - | - | ? |
| ZPAL V.39/394 | R | - | - | - | - | - | - | - | - | - | - | - | - | - | - | - | - | - | - | - | - | - | - | - | - | - | - | - | ? |
| | L | - | P | - | - | - | - | P | - | - | - | - | - | - | - | - | P | + | P | - | - | - | - | - | - | - | - | - | ? |
| ZPAL V.39/395 | R | - | - | - | - | - | - | - | - | - | - | - | - | - | - | - | - | - | - | - | - | - | - | - | - | - | - | - | ? |
| | L | - | P | - | - | - | - | P | - | - | - | - | - | - | - | - | P | + | P | - | - | - | - | - | - | - | - | - | ? |
| ZPAL V.39/396 | R | - | - | - | - | - | - | - | - | - | - | - | - | - | - | - | - | - | - | - | - | - | - | - | - | - | - | - | ? |
| | L | - | P | P | P | - | - | + | P | - | - | P | - | - | - | P | + | + | P | P | - | - | - | - | - | - | - | - | ? |
| ZPAL V.39/397 | R | - | - | - | - | - | - | - | - | - | - | - | - | - | - | - | - | - | - | - | - | - | - | - | - | - | - | - | ? |
| | L | - | - | - | - | - | - | - | - | - | - | - | - | - | - | - | - | - | - | - | - | - | + | - | - | - | - | - | ? |
| ZPAL V.39/399 | R | - | - | - | - | - | - | - | - | - | - | - | - | - | - | - | - | - | - | - | - | - | - | - | - | - | - | ?+ | ?X |
| | L | - | - | - | - | - | - | - | - | - | - | - | - | - | - | - | - | - | - | - | - | - | - | - | - | - | - | - | ? |
| ZPAL V.39/400 | R | - | - | - | - | - | - | - | - | - | - | - | - | - | - | - | - | - | - | - | - | - | - | - | - | - | - | - | ? |
| | L | - | - | - | - | - | - | - | - | - | - | - | - | + | - | - | - | - | - | - | - | + | P | - | - | - | - | - | ? |
| ZPAL V.39/402 | R | - | - | - | - | - | P | - | - | - | - | - | - | - | - | - | - | - | - | - | - | - | - | - | - | - | - | - | ? |
| | L | - | - | - | - | - | P | - | - | - | - | - | - | - | - | - | - | - | - | - | - | - | - | - | - | - | - | - | ? |

**Table S4.** *Proterochersis porebensis*, plastron scutes. **+** – preserved whole or nearly whole; **P** – partially preserved; **-** – not preserved or insignificant part preserved. **R** means right, **L** means left side of the body.

| Specimen | Side | Extragular | Gular | Humeral | Pectoral | Abdominal | | Femoral | Anal | Caudal | Intercaudal | Axillary | Inframarginal | | | |
|---|---|---|---|---|---|---|---|---|---|---|---|---|---|---|---|---|
| | | | | | | 1 | 2 | | | | | | 1 | 2 | 3 | 4 |
| ZPAL V.39/8 | R | - | - | - | - | - | - | - | - | - | - | - | - | - | - | - |
| | L | - | - | - | - | - | - | - | - | - | | - | P | P | P | - |
| ZPAL V.39/13 | R | - | - | - | - | - | - | P | P | - | P | - | - | - | - | - |
| | L | - | - | - | - | - | | P | P | - | | - | - | - | - | - |
| ZPAL V.39/21 | R | - | - | - | - | - | - | - | - | - | - | - | P | P | - | - |
| | L | - | - | - | - | - | - | - | - | - | | - | - | - | - | - |
| ZPAL V.39/34 | R | - | - | P | P | P | P | P | P | + | + | P | P | P | P | P |
| | L | + | P | P | P | P | P | P | + | P | | P | + | + | + | P |
| ZPAL V.39/48 | R | P | P | P | + | + | + | + | + | P | + | + | + | + | + | + |
| | L | + | + | P | + | + | + | + | + | + | | P | + | + | P | + |
| ZPAL V.39/49 | R | + | + | + | + | P | P | + | + | P | + | P | + | + | + | + |
| | L | P | + | + | + | + | + | + | + | + | | + | P | + | + | P |
| ZPAL V.39/56 | R | - | - | - | - | - | - | - | - | - | - | - | - | - | - | - |
| | L | - | - | - | - | - | - | - | - | + | | - | - | - | - | - |
| ZPAL V.39/62 | R | - | - | - | P | - | - | - | - | - | - | - | - | - | - | - |
| | L | - | - | - | P | - | - | - | - | - | | - | - | - | - | - |
| ZPAL V.39/64 | R | - | - | - | - | - | - | - | - | - | - | - | - | - | - | - |
| | L | - | - | P | P | - | - | - | - | - | | - | - | - | - | - |
| ZPAL V.39/66 | R | - | - | - | - | - | - | - | P | P | P | - | - | - | - | - |
| | L | - | - | - | - | - | - | - | P | + | | - | - | - | - | - |
| ZPAL V.39/68 | R | - | - | - | - | - | - | - | P | P | + | - | - | - | - | - |
| | L | - | - | - | - | - | - | - | P | P | | - | - | - | - | - |
| ZPAL V.39/69 | R | - | - | - | - | - | - | - | + | + | + | - | - | - | - | - |
| | L | - | - | - | - | - | - | - | + | + | | - | - | - | - | - |
| ZPAL V.39/70 | R | - | - | - | - | - | - | - | P | + | + | - | - | - | - | - |
| | L | - | - | - | - | - | - | - | P | P | | - | - | - | - | - |
| ZPAL V.39/71 | R | - | - | - | - | - | - | - | P | + | + | - | - | - | - | - |
| | L | - | - | - | - | - | - | - | P | P | | - | - | - | - | - |
| ZPAL V.39/157 | R | - | - | - | - | - | - | P | P | - | - | - | - | - | - | - |
| | L | - | - | - | - | - | - | - | - | - | | - | - | - | - | - |
| ZPAL V.39/159 | R | - | - | - | P | P | P | P | - | - | - | - | - | - | - | - |
| | L | - | - | - | - | - | - | - | - | - | | - | - | - | - | - |
| ZPAL V.39/160 | R | - | - | - | - | - | - | - | - | - | - | - | - | P | P | P |
| | L | - | - | - | - | - | - | - | - | - | | - | - | - | - | - |
| ZPAL V.39/170 | R | - | - | - | - | - | - | - | - | - | - | - | - | - | - | - |
| | L | - | - | - | - | - | - | P | P | - | | - | - | - | - | - |
| ZPAL V.39/172 | R | - | - | P | - | - | - | - | - | - | - | - | - | - | - | - |
| | L | - | - | P | - | - | - | - | - | - | | - | - | - | - | - |
| ZPAL V.39/185 | R | - | - | - | - | - | - | - | - | - | - | - | - | - | - | - |
| | L | - | - | - | P | - | - | - | - | - | | - | - | - | - | - |
| ZPAL V.39/186 | R | - | - | - | - | - | - | - | - | - | - | - | - | - | - | - |
| | L | - | P | - | - | - | - | - | - | - | | - | - | - | - | - |
| ZPAL V.39/187 | R | + | P | - | - | - | - | - | - | - | - | - | - | - | - | - |
| | L | - | - | - | - | - | - | - | - | - | | - | - | - | - | - |

| Specimen | Side | Extragular | Gular | Humeral | Pectoral | Abdominal | | Femoral | Anal | Caudal | Intercaudal | Axillary | Inframarginal | | | |
|---|---|---|---|---|---|---|---|---|---|---|---|---|---|---|---|---|
| | | | | | | 1 | 2 | | | | | | 1 | 2 | 3 | 4 |
| ZPAL V.39/189 | R | P | P | - | - | - | - | - | - | - | - | - | - | - | - | - |
| | L | - | - | - | - | - | - | - | - | - | | - | - | - | - | - |
| ZPAL V.39/194 | R | - | - | P | P | - | - | - | - | - | - | - | - | - | - | - |
| | L | - | - | - | - | - | - | - | - | - | | - | - | - | - | - |
| ZPAL V.39/199 | R | - | - | - | - | - | - | - | - | - | - | - | - | - | - | - |
| | L | - | - | - | - | - | - | - | - | + | | - | - | - | - | - |
| ZPAL V.39/200 | R | - | - | - | - | - | - | - | - | - | - | - | - | - | - | - |
| | L | - | - | - | - | - | - | - | - | ?+ | | - | - | - | - | - |
| ZPAL V.39/333 | R | + | P | P | - | - | - | - | - | - | - | - | - | - | - | - |
| | L | - | - | P | - | - | - | - | - | - | | - | - | - | - | - |
| ZPAL V.39/343 | R | - | - | - | - | - | - | - | - | - | - | - | - | - | - | - |
| | L | - | - | - | P | P | P | - | - | - | | - | - | - | - | - |
| ZPAL V.39/379 | R | + | + | P | - | - | - | - | - | - | - | - | - | - | - | - |
| | L | - | - | - | - | - | - | - | - | - | | - | - | - | - | - |
| ZPAL V.39/385 | R | P | + | P | P | P | - | - | - | - | - | - | - | - | - | - |
| | L | + | + | P | P | P | - | - | - | - | | - | - | - | - | - |
| ZPAL V.39/387 | R | - | - | - | - | - | - | - | - | - | - | - | - | - | - | - |
| | L | + | + | P | - | - | - | - | - | - | | - | - | - | - | - |
| ZPAL V.39/388 | R | P | P | - | - | - | - | - | - | - | - | - | - | - | - | - |
| | L | - | - | - | - | - | - | - | - | - | | - | - | - | - | - |
| ZPAL V.39/389 | R | - | - | - | - | - | - | - | - | - | - | - | - | - | - | - |
| | L | - | - | - | - | - | - | - | - | + | | - | - | - | - | - |
| ZPAL V.39/400 | R | - | - | - | - | - | - | - | - | - | - | - | - | - | - | - |
| | L | - | - | - | - | - | - | - | - | - | | - | - | - | P | - |
| ZPAL V.39/404 | R | P | P | - | - | - | - | - | - | - | - | - | - | - | - | - |
| | L | - | - | - | - | - | - | - | - | - | | - | - | - | - | - |
| ZPAL V.39/420 | R | - | - | - | - | - | - | - | - | - | - | - | - | - | - | - |
| | L | + | + | P | - | - | - | - | - | - | | - | - | - | - | - |

**Table S5.** Measurements of *Proterochersis* spp. given in the text.

| # | | Neural canal | | | | Carapace | | | | | | | | Plastron | | | | | Ilium |
|---|---|---|---|---|---|---|---|---|---|---|---|---|---|---|---|---|---|---|---|
| | | Dorsal | | Sacral | | Complete length | Cervical scute | Supramarginal scute | | Ninth marginal | Caudalmost marginal scute | | | Gular | Extragular | Intercaudal scute | | Femoral scute area | Width |
| | | Height | Width | Height | Width | | Length | Height | Length | Length | Width | Length | Max size | Width | Width | Length | Width | Thickness | |
| ZPAL | V.39/8 | | | | | | | 5.5 | 8.0 | | | | | | | | | | |
| | V.39/18 | | | | | | | | | | 2.1 | 2.0 | 2.4 | | | | | | |
| | V.39/22 | | | | | | 1.5 | | | | | | | | | | | | |
| | V.39/23 | | | | | | | | | | 1.6 | 1.4 | 1.9 | | | | | | |
| | V.39/34 | | | | | ~28.0 | 0.8 | | ~4.2 | 3.5 | 2.1 | 1.0 | | | | | | 1.8 | ~2.0 |
| | V.39/48 | ~0.6 | ~0.7 | | | ~42.5 | 1.5 | 4.2 | 6.6 | 5.2 | | | | 2.6 | 3.2 | | | 2.1 | 3.5 |
| | V.39/49 | ~0.8 | ~0.7 | | | ~49.0 | 2.2 | 5.0 | 7.7 | 6.5 | | | | 2.7/2.4 | | | | 2.5 | 4.0 |
| | V.39/57 | | | | | | 2.4 | | | | | | | | | | | | |
| | V.39/59 | | | | | | | | | | 3.2 | 3.9 | 5.1 | | | | | | |
| | V.39/60 | | | | | | | | | 7.8 | | | | | | | | | |
| | V.39/63 | | | | | | | | | | | | | | | | | | 6.3 |
| | V.39/68 | | | | | | | | | | | | | | | 2.2 | 1.2 | | |
| | V.39/69 | | | | | | | | | | | | | | | 2.3 | 2.2 | | |
| | V.39/72 | | | | | ~44.5 | 1.9 | 4.2 | 6.7 | 6.0 | | | | | | | | | 3.7 |
| | V.39/157 | | | | | | | | | | | | | | | | | 2.7 | |
| | V.39/169 | ~0.4 | ~0.5 | | | | | | | | | | | | | | | | |
| | V.39/370 | | | 0.8 | 0.7 | | | | | | | | | | | | | | |
| | V.39/377 | 0.8 | ~0.3 | | | | | | | | | | | | | | | | |
| | V.39/378 | ~0.4 | ~0.5 | | | | | | | | | | | | | | | | |
| | V.39/385 | | | | | | | | | | | | | 1.9 | 3.9 | | | | |
| | V.39/390 | | | | | | 1.0 | | | | | | | | | | | | |
| | V.39/402 | ~0.8 | ~1.0 | 1.0 | 0.9 | | | | | | | | | | | | | | |
| SMNS | 17561 | | | | | ~35.0 | | | | | | | | | | | | | |
| | 17930 | | | | | ~36.0 | | | | | | | | | | | | | |
| CSMM | uncat. | | | | | 36.5 | | | | | | | | | | | | | |

**Table S6.** MANOVA of size variation in the anal plastral region.

| Effect | SS | MS | df | F | p-value |
|---|---|---|---|---|---|
| **Species (centroid size)** | 275.08 | 275.08 | 1 | 2.8 | > 0.3 |
| **Species (shape)** | 0.01 | 0.002 | 30 | 0.32 | 1 |
| **Sex (centroid size)** | 284.67 | 284.67 | 1 | 3.21 | > 0.3 |
| **Sex (shape)** | 0.04 | 0.001 | 30 | 2.48 | < 0.01 |

**Table S7.** Examples of sexual dimorphism in extant and fossil turtles.

| Body section | | Feature | References |
|---|---|---|---|
| **Limbs** | | Feet wideness | Keswick & Hofmeyr 2015 |
| | | Rugose surfaces on male hindlimbs | Berry & Shine 1980 |
| | | Size and shape of claws on forelimbs | Wibbels *et al.* 1991; Wyneken 2001 |
| **Pelvis** | | Size of pelvic aperture | Cordero 2018 |
| **Shell** | **Whole shell** | Kinesis | Pritchard 2008; Keswick & Hofmeyr 2015 |
| | | Presence or absence of particular shell scutes | Lichtig & Lucas 2017 |
| | | Relative shape and size of particular shell scutes or bones | Brophy 2006; Leuteritz & Gantz 2013; Lichtig & Lucas 2017 |
| | | Shape and size of caudal aperture of the shell | Pritchard 2008; Leuteritz & Gantz 2013; Keswick & Hofmeyr 2015 |
| | | Shell height | Brophy 2006; Pritchard 2008; Keswick & Hofmeyr 2015 |
| | | Shell length | Chiari & Claude 2011 |
| | | Shell width | Brophy 2006; Chiari & Claude 2011; Keswick & Hofmeyr 2015 |
| | | Time of fontanelle retention | Pritchard 2008 |
| | **Carapace** | Allometric growth dynamic | Chiari & Claude 2011; Keswick & Hofmeyr 2015 |
| | | Degree of saddling | Pritchard 2008 |
| | | Shape of the caudal part | Wibbels *et al.* 1991; Pritchard 2008 |
| | | Plastron length | Brophy 2006 |
| | **Plastron** | Seasonal plastron de-ossification and scute softening in males | Wibbels *et al.* 1991; Wyneken 2001; Pritchard 2008 |
| | | Shape and size of anal notch | Brophy 2006; Pritchard 2008; Cadena *et al.* 2013; Leuteritz & Gantz 2013; Sullivan & Joyce 2017 |
| | | Shape and size of gular processes | Pritchard 2008 |
| | | Ventral concavity | Wyneken 2001; Pritchard 2008; Cadena *et al.* 2013; Leuteritz & Gantz 2013 |
| **Tail** | | Cloacal position | White & Murphy 1973; Wyneken 2001 |
| | | Tail length | Berry & Shine 1980; Wibbels *et al.* 1991; Wyneken 2001; Sullivan & Joyce 2017 |
| **Whole body** | | Body size | Berry & Shine 1980; Brophy 2006; Pritchard 2008; Leuteritz & Gantz 2013; Ceballos & Iverson 2014; Keswick & Hofmeyr 2015; Lichtig & Lucas 2017; Cordero 2018 |

# PRINCIPAL COMPONENT ANALYSIS

## PRINCIPAL COMPONENT COEFFICIENTS FOR GULARS AND EXTRAGULARS IN VENTRAL VIEW

| | PC1 | PC2 | PC3 | PC4 | PC5 | PC6 | PC7 | PC8 | PC9 | PC10 |
|---|---|---|---|---|---|---|---|---|---|---|
| x1 | 0,088652 | -0,232026 | -0,000252 | 0,070683 | -0,009663 | 0,309625 | -0,368513 | 0,095648 | -0,073229 | 0,355246 |
| y1 | 0,526482 | 0,291836 | 0,109098 | -0,403410 | -0,094228 | -0,133440 | 0,113118 | -0,383049 | -0,103834 | 0,021558 |
| x2 | -0,407408 | -0,108021 | 0,476158 | -0,565403 | 0,173562 | 0,086573 | -0,260376 | -0,094146 | -0,193022 | -0,183285 |
| y2 | 0,193091 | 0,161074 | -0,182779 | -0,270955 | -0,319280 | 0,498776 | -0,007306 | 0,372947 | -0,149040 | -0,130456 |
| x3 | 0,164788 | 0,169812 | 0,399969 | 0,399016 | -0,194046 | 0,090516 | -0,049367 | 0,071571 | -0,208998 | -0,241876 |
| y3 | -0,065301 | 0,382683 | 0,037788 | 0,051472 | 0,139891 | 0,002903 | 0,015141 | -0,036708 | 0,050137 | 0,090778 |
| x4 | 0,212117 | 0,107146 | 0,178894 | 0,128081 | 0,011423 | 0,127594 | -0,166298 | -0,189403 | 0,035066 | -0,028307 |
| y4 | -0,002224 | 0,287338 | 0,013334 | 0,022336 | 0,126198 | -0,053230 | -0,034848 | 0,001677 | 0,092070 | -0,010319 |
| x5 | 0,199756 | 0,009757 | -0,111636 | -0,099171 | 0,141994 | 0,098174 | -0,246033 | -0,161771 | 0,237931 | 0,284321 |
| y5 | 0,059485 | 0,149784 | -0,099097 | -0,039773 | 0,127787 | -0,059255 | -0,132162 | 0,075852 | 0,184739 | -0,007018 |
| x6 | 0,125275 | -0,111971 | -0,189464 | -0,167777 | 0,216657 | -0,042881 | 0,083680 | 0,216889 | -0,173045 | 0,092700 |
| y6 | 0,076106 | -0,049135 | -0,163081 | -0,107771 | 0,172912 | -0,142463 | 0,063994 | 0,354939 | -0,178633 | -0,096555 |
| x7 | 0,045370 | -0,124813 | -0,141129 | -0,129049 | 0,062868 | -0,112283 | 0,130274 | 0,068325 | -0,073588 | -0,081593 |
| y7 | 0,026773 | -0,220678 | -0,057543 | -0,039290 | -0,099572 | -0,335497 | 0,004971 | -0,072574 | -0,035726 | -0,302317 |
| x8 | -0,014543 | -0,054915 | -0,112996 | -0,107937 | 0,033948 | -0,062654 | 0,145989 | 0,067787 | -0,029008 | 0,012802 |
| y8 | 0,009132 | -0,284785 | 0,080092 | -0,006906 | -0,331489 | -0,232842 | 0,038619 | -0,074130 | 0,180521 | 0,053521 |
| x9 | -0,073234 | 0,025245 | -0,095842 | -0,084649 | 0,036437 | -0,043623 | 0,158444 | 0,007494 | 0,078930 | -0,032515 |
| y9 | -0,010290 | -0,265237 | 0,133975 | -0,031920 | -0,292313 | -0,034595 | 0,094678 | -0,013125 | -0,062192 | 0,429401 |
| x10 | -0,127384 | 0,084728 | -0,061865 | -0,062693 | 0,029616 | -0,018424 | 0,139605 | 0,019388 | 0,166387 | 0,029940 |
| y10 | -0,042742 | -0,173606 | 0,086308 | 0,000081 | -0,193245 | 0,104120 | 0,216000 | -0,134971 | -0,174810 | 0,138532 |
| x11 | -0,177274 | 0,136425 | -0,022345 | -0,054519 | -0,052394 | 0,031003 | 0,148821 | -0,015158 | 0,215720 | 0,063791 |

| | | | | | | | | | | |
|---|---|---|---|---|---|---|---|---|---|---|
| y11 | -0,098610 | -0,069154 | 0,040821 | 0,069246 | 0,030120 | 0,163371 | 0,222184 | -0,137254 | -0,116342 | 0,139279 |
| x12 | -0,238601 | 0,202936 | 0,019605 | -0,053355 | -0,161443 | 0,029534 | 0,202701 | 0,004126 | 0,224311 | 0,099598 |
| y12 | -0,126783 | -0,015576 | -0,010659 | 0,107174 | 0,324507 | 0,234917 | 0,260407 | -0,188978 | 0,002340 | 0,105546 |
| x13 | -0,188436 | 0,174166 | -0,030639 | 0,081465 | -0,215392 | -0,001357 | 0,143313 | 0,070196 | -0,046986 | -0,004940 |
| y13 | -0,151196 | -0,014219 | -0,146242 | 0,184023 | 0,145591 | 0,231830 | 0,106389 | -0,158528 | -0,204659 | -0,112006 |
| x14 | -0,125966 | 0,135489 | -0,083096 | 0,075270 | -0,200941 | -0,133232 | -0,053249 | 0,012502 | -0,197652 | -0,008164 |
| y14 | -0,139794 | -0,028551 | -0,277381 | 0,133419 | 0,019474 | 0,072092 | -0,179122 | -0,226456 | -0,270503 | -0,173035 |
| x15 | -0,066966 | 0,100332 | -0,059189 | 0,061068 | -0,146606 | -0,135229 | -0,098403 | 0,046012 | -0,042050 | -0,021187 |
| y15 | -0,117663 | -0,048044 | -0,287110 | -0,014418 | -0,064964 | 0,005281 | -0,311132 | -0,229172 | 0,145738 | -0,038685 |
| x16 | -0,026336 | 0,062688 | -0,064262 | 0,068261 | -0,056993 | -0,166158 | -0,054208 | -0,037018 | -0,119460 | 0,007428 |
| y16 | -0,074953 | -0,074855 | -0,071675 | -0,000523 | -0,146443 | 0,079553 | -0,238234 | 0,060671 | 0,353100 | -0,212687 |
| x17 | 0,012973 | 0,029305 | -0,103948 | 0,083696 | -0,014737 | -0,167933 | -0,111423 | -0,050608 | -0,066841 | 0,115972 |
| y17 | -0,046999 | -0,070520 | 0,103441 | 0,010563 | -0,051228 | 0,071971 | -0,055222 | 0,118373 | 0,188665 | -0,202149 |
| x18 | 0,062588 | -0,043990 | -0,090583 | 0,086413 | 0,016006 | -0,094946 | -0,084216 | 0,040053 | -0,077431 | 0,076613 |
| y18 | -0,027713 | -0,025099 | 0,164498 | 0,039638 | 0,029208 | -0,010659 | 0,009798 | 0,084869 | 0,170696 | -0,059257 |
| x19 | 0,118360 | -0,122058 | -0,045243 | 0,090122 | 0,064916 | -0,012749 | -0,013532 | -0,088345 | 0,006972 | -0,115515 |
| y19 | -0,012367 | 0,007647 | 0,191335 | 0,068998 | 0,095921 | -0,086806 | 0,011775 | 0,203998 | 0,084651 | 0,102706 |
| x20 | 0,169532 | -0,193386 | 0,027983 | 0,099881 | 0,125477 | 0,054274 | 0,131577 | -0,181474 | 0,082487 | -0,223731 |
| y20 | 0,015001 | 0,031104 | 0,181445 | 0,091189 | 0,148609 | -0,131421 | -0,083652 | 0,300145 | 0,002034 | 0,176959 |
| x21 | 0,246737 | -0,246849 | 0,109878 | 0,080598 | 0,139310 | 0,164175 | 0,221214 | 0,097932 | 0,253508 | -0,197299 |
| y21 | 0,010565 | 0,027993 | 0,153430 | 0,136826 | 0,232544 | -0,244608 | -0,115396 | 0,081473 | -0,158949 | 0,086201 |

**PRINCIPAL COMPONENT COEFFICIENTS FOR SECTIONED GULARS**

| | PC1 | PC2 | PC3 | PC4 | PC5 | PC6 | PC7 | PC8 | PC9 |
|---|---|---|---|---|---|---|---|---|---|
| x1 | 0,249798 | -0,259061 | 0,153091 | -0,467814 | 0,055956 | 0,272172 | 0,029684 | -0,157621 | 0,154001 |
| y1 | -0,175060 | -0,314140 | -0,065367 | -0,288720 | -0,061554 | 0,312190 | -0,142677 | 0,189876 | 0,221518 |
| x2 | 0,179387 | -0,275286 | 0,315104 | 0,076572 | 0,259868 | -0,107752 | 0,213988 | 0,048680 | -0,372785 |
| y2 | -0,187546 | -0,277455 | 0,063426 | 0,163951 | 0,144338 | -0,076013 | 0,086587 | 0,303936 | -0,080143 |
| x3 | 0,041577 | -0,174545 | 0,319421 | 0,229572 | 0,057046 | -0,202093 | -0,033695 | -0,233790 | 0,140636 |
| y3 | -0,245260 | -0,124884 | 0,062990 | 0,238973 | -0,011293 | -0,098310 | 0,072000 | 0,022999 | 0,158799 |
| x4 | -0,126645 | 0,008961 | 0,249078 | 0,154860 | -0,240526 | -0,009970 | -0,313383 | 0,148576 | 0,342905 |
| y4 | -0,317561 | 0,037876 | 0,028003 | 0,165920 | -0,114249 | 0,040339 | -0,169594 | -0,241112 | -0,110521 |
| x5 | -0,231825 | 0,197545 | 0,105751 | -0,010321 | 0,013165 | 0,268691 | 0,166287 | 0,347084 | -0,077176 |
| y5 | -0,343368 | 0,189343 | -0,043327 | 0,096082 | 0,059270 | 0,139037 | 0,063662 | -0,277270 | -0,220795 |
| x6 | -0,174480 | 0,186909 | -0,008890 | -0,134190 | 0,194361 | 0,082448 | -0,057016 | 0,047427 | 0,106756 |
| y6 | -0,143134 | 0,213362 | -0,239421 | -0,457194 | 0,148389 | -0,207837 | 0,254351 | -0,111475 | 0,086368 |
| x7 | -0,163665 | 0,092875 | -0,045247 | 0,049410 | -0,003834 | 0,109757 | 0,046826 | -0,192328 | 0,245706 |
| y7 | 0,189729 | -0,015302 | -0,210865 | -0,120624 | -0,077541 | -0,462366 | -0,435544 | 0,165590 | -0,078919 |
| x8 | -0,208554 | -0,052903 | -0,100819 | -0,150991 | 0,130665 | -0,141248 | -0,160874 | 0,276102 | -0,376598 |
| y8 | 0,264903 | 0,026468 | -0,144641 | 0,167055 | -0,294826 | 0,003110 | 0,221259 | 0,019636 | 0,122755 |
| x9 | -0,154291 | -0,148789 | -0,092985 | -0,111654 | -0,140047 | -0,323397 | -0,094160 | -0,214265 | -0,033689 |
| y9 | 0,206376 | 0,017974 | -0,122851 | 0,123074 | -0,126113 | 0,113049 | 0,113280 | 0,133807 | 0,064796 |
| x10 | -0,055219 | -0,239557 | -0,223263 | 0,040573 | -0,332683 | -0,094670 | 0,400760 | -0,089784 | -0,063957 |
| y10 | 0,169517 | -0,013990 | -0,057552 | 0,065740 | -0,078856 | 0,175467 | 0,064654 | 0,264714 | -0,119678 |
| x11 | 0,049358 | -0,152508 | -0,234610 | 0,012516 | -0,167382 | -0,032062 | 0,146706 | -0,031562 | 0,042920 |
| y11 | 0,146969 | -0,055624 | -0,037402 | 0,058398 | 0,027188 | 0,159450 | -0,144880 | -0,073102 | -0,102578 |

| | | | | | | | | | |
|---|---|---|---|---|---|---|---|---|---|
| x12 | 0,134120 | -0,034908 | -0,190347 | -0,034974 | 0,041526 | 0,175999 | -0,191735 | -0,132714 | -0,177708 |
| y12 | 0,147703 | -0,067740 | -0,000307 | 0,037145 | 0,130575 | 0,142605 | -0,229762 | -0,344544 | -0,094254 |
| x13 | 0,144524 | 0,141328 | -0,166672 | 0,174833 | 0,176762 | 0,099822 | -0,185988 | 0,203974 | 0,140689 |
| y13 | 0,121748 | 0,011767 | 0,044579 | 0,115062 | 0,318750 | 0,093951 | -0,004598 | -0,144683 | 0,049267 |
| x14 | 0,151631 | 0,297459 | -0,109926 | 0,103324 | 0,165888 | -0,206545 | 0,010839 | 0,035696 | 0,255414 |
| y14 | 0,098094 | 0,131808 | 0,163149 | -0,051668 | 0,321613 | -0,227939 | 0,267906 | 0,056046 | 0,181288 |
| x15 | 0,164283 | 0,412481 | 0,030314 | 0,068284 | -0,210768 | 0,108846 | 0,021760 | -0,055477 | -0,327115 |
| y15 | 0,066889 | 0,240537 | 0,559589 | -0,313195 | -0,385689 | -0,106734 | -0,016643 | 0,035581 | -0,077904 |

**Principal Component Coefficients for caudal processes**

| | PC1 | PC2 | PC3 | PC4 | PC5 | PC6 | PC7 | PC8 | PC9 |
|---|---|---|---|---|---|---|---|---|---|
| x1 | -0,207782 | 0,029857 | -0,301730 | -0,307655 | 0,157363 | -0,395922 | 0,072367 | -0,011466 | 0,320984 |
| y1 | 0,029789 | 0,359727 | -0,499377 | 0,092208 | -0,067751 | -0,217315 | -0,283477 | -0,194601 | -0,228382 |
| x2 | -0,032834 | -0,097825 | -0,154618 | 0,283460 | -0,621150 | 0,086964 | 0,466157 | 0,139665 | -0,076186 |
| y2 | 0,243620 | -0,009999 | 0,006578 | 0,247318 | -0,110652 | 0,022416 | -0,309677 | 0,378158 | 0,536441 |
| x3 | -0,048253 | -0,271796 | -0,342903 | -0,089378 | 0,227227 | 0,586215 | -0,280985 | -0,041611 | 0,138337 |
| y3 | 0,050755 | 0,210506 | -0,038782 | -0,191148 | 0,408175 | 0,323608 | 0,512919 | 0,293728 | -0,112104 |
| x4 | 0,036343 | -0,186036 | 0,224554 | -0,014185 | -0,037690 | 0,012032 | -0,212749 | 0,154020 | -0,073443 |
| y4 | 0,287944 | -0,163553 | 0,024638 | -0,002427 | 0,317649 | -0,306591 | 0,211895 | -0,101112 | -0,041099 |
| x5 | -0,008120 | -0,164547 | 0,209540 | 0,032387 | 0,079531 | -0,159213 | -0,131625 | 0,010078 | -0,134472 |
| y5 | 0,182413 | -0,171842 | -0,004430 | -0,080125 | -0,022777 | 0,128883 | 0,052402 | -0,122152 | 0,155065 |
| x6 | -0,047714 | -0,113773 | 0,200771 | 0,093054 | 0,118659 | -0,180322 | -0,058923 | 0,051573 | -0,087532 |
| y6 | 0,071417 | -0,220109 | -0,029735 | -0,154160 | -0,149886 | 0,188582 | 0,003773 | -0,347205 | -0,054637 |
| x7 | -0,090798 | -0,048256 | 0,183757 | 0,122171 | 0,127668 | -0,054305 | -0,010572 | 0,000975 | -0,049269 |

| | | | | | | | | | |
|---|---|---|---|---|---|---|---|---|---|
| y7 | -0,045880 | -0,306138 | -0,073875 | -0,237172 | -0,202147 | -0,004879 | -0,016931 | -0,255550 | -0,146631 |
| x8 | -0,140882 | -0,000522 | 0,132728 | 0,122452 | 0,098064 | 0,005670 | -0,043047 | -0,201670 | 0,068633 |
| y8 | -0,143278 | -0,339197 | -0,058433 | -0,240785 | -0,157937 | -0,183273 | 0,070474 | 0,214901 | -0,014782 |
| x9 | -0,197209 | -0,004120 | 0,083227 | 0,115181 | 0,073861 | 0,013628 | 0,011859 | -0,223877 | -0,035019 |
| y9 | -0,233891 | -0,221903 | -0,109142 | -0,044119 | 0,080167 | -0,167246 | -0,081764 | 0,420620 | -0,151396 |
| x10 | -0,240640 | 0,043488 | 0,021680 | 0,125692 | 0,035022 | 0,092445 | 0,112701 | 0,043834 | -0,034437 |
| y10 | -0,312570 | 0,083774 | -0,125447 | 0,273968 | 0,050331 | 0,013546 | -0,035090 | 0,014363 | -0,133104 |
| x11 | -0,161813 | 0,160927 | 0,130070 | -0,101837 | 0,000242 | 0,148690 | 0,015853 | -0,063527 | 0,013119 |
| y11 | -0,208316 | 0,124329 | -0,024636 | 0,184283 | 0,029700 | 0,079774 | -0,042868 | -0,076927 | -0,027803 |
| x12 | -0,040710 | 0,220483 | 0,054514 | -0,279792 | -0,207219 | -0,004411 | 0,054834 | 0,037610 | 0,273518 |
| y12 | -0,119499 | 0,105978 | 0,016467 | 0,155713 | 0,019129 | -0,008055 | 0,041639 | -0,036849 | 0,136046 |
| x13 | 0,057329 | 0,237797 | 0,012071 | -0,281496 | -0,123493 | -0,067324 | 0,033404 | -0,077334 | 0,191615 |
| y13 | -0,053129 | 0,082200 | 0,053068 | 0,151656 | 0,021959 | 0,015330 | 0,022663 | 0,004787 | 0,109352 |
| x14 | 0,154782 | 0,190436 | -0,071007 | -0,181523 | -0,060707 | -0,037194 | -0,078422 | 0,098368 | -0,185343 |
| y14 | -0,000475 | 0,076904 | 0,111247 | 0,103619 | 0,006829 | 0,025287 | 0,023845 | -0,046715 | 0,147405 |
| x15 | 0,237261 | 0,125051 | -0,126472 | -0,016025 | -0,012375 | 0,057709 | -0,146645 | 0,169538 | -0,267092 |
| y15 | 0,050435 | 0,073306 | 0,188216 | 0,009604 | -0,032753 | -0,028460 | 0,078809 | -0,133639 | 0,155252 |
| x16 | 0,324696 | -0,001033 | -0,135142 | 0,109516 | 0,029032 | -0,004783 | 0,002544 | 0,112301 | -0,093703 |
| y16 | 0,082577 | 0,133905 | 0,262269 | -0,072925 | -0,050109 | 0,007099 | -0,068100 | -0,125711 | -0,043809 |
| x17 | 0,406343 | -0,120131 | -0,121039 | 0,267979 | 0,115966 | -0,099877 | 0,193250 | -0,198477 | 0,030289 |
| y17 | 0,118088 | 0,182112 | 0,301373 | -0,195509 | -0,139928 | 0,111294 | -0,180513 | 0,113901 | -0,285815 |

### RESULTS OF REGRESSION ANALYSIS FOR GULARS AND EXTRAGULARS IN VENTRAL VIEW

Total sample size: 11

Regression Coefficients

| | | | | | |
|---|---|---|---|---|---|
| ProcCoord1 | 0,05820842 | ProcCoord15 | 0,01295491 | ProcCoord29 | -0,01839421 |
| ProcCoord2 | 0,02293745 | ProcCoord16 | 0,02734338 | ProcCoord30 | 0,03176098 |
| ProcCoord3 | -0,04760614 | ProcCoord17 | -0,00700109 | ProcCoord31 | -0,00970878 |
| ProcCoord4 | 0,03843660 | ProcCoord18 | 0,02056299 | ProcCoord32 | 0,01704538 |
| ProcCoord5 | -0,04867311 | ProcCoord19 | -0,02484100 | ProcCoord33 | 0,00397286 |
| ProcCoord6 | -0,06686331 | ProcCoord20 | 0,00919663 | ProcCoord34 | -0,00322649 |
| ProcCoord7 | -0,00008640 | ProcCoord21 | -0,04041714 | ProcCoord35 | 0,01884070 |
| ProcCoord8 | -0,04304497 | ProcCoord22 | -0,00971369 | ProcCoord36 | -0,01836300 |
| ProcCoord9 | 0,04567955 | ProcCoord23 | -0,06239325 | ProcCoord37 | 0,03315441 |
| ProcCoord10 | -0,00239963 | ProcCoord24 | -0,01733141 | ProcCoord38 | -0,02817712 |
| ProcCoord11 | 0,04700084 | ProcCoord25 | -0,05038202 | ProcCoord39 | 0,03988737 |
| ProcCoord12 | 0,02545187 | ProcCoord26 | -0,00386046 | ProcCoord40 | -0,02725621 |
| ProcCoord13 | 0,03251643 | ProcCoord27 | -0,02882654 | ProcCoord41 | 0,04611421 |
| ProcCoord14 | 0,03348881 | ProcCoord28 | 0,01966098 | ProcCoord42 | -0,02564876 |

Sums of squares (the sums of squares below are within-group SSs)

Total SS: 0,09200218

Predicted SS: 0,01593230

Residual SS: 0,07606988

% predicted: 17,3173%

Permutation test against the null hypothesis of independence

Number of randomization rounds: 10000

P-value: 0,1207

### RESULTS OF REGRESSION ANALYSIS FOR CROSS-SECTIONED GULARS

Total sample size: 10

Regression Coefficients

| | | | | | |
|---|---|---|---|---|---|
| ProcCoord1 | 0,00127316 | ProcCoord7 | 0,00552270 | ProcCoord13 | 0,00335539 |
| ProcCoord2 | -0,03187105 | ProcCoord8 | -0,01065635 | ProcCoord14 | -0,00751846 |
| ProcCoord3 | -0,02036913 | ProcCoord9 | 0,01679392 | ProcCoord15 | -0,02737244 |
| ProcCoord4 | -0,04474181 | ProcCoord10 | 0,00114301 | ProcCoord16 | 0,01771224 |
| ProcCoord5 | -0,01465551 | ProcCoord11 | 0,01009951 | ProcCoord17 | -0,03473907 |
| ProcCoord6 | -0,02947083 | ProcCoord12 | -0,00084811 | ProcCoord18 | 0,01548106 |

| | | | | | |
|---|---|---|---|---|---|
| ProcCoord19 | -0,03511381 | ProcCoord23 | 0,00512285 | ProcCoord27 | 0,02892643 |
| ProcCoord20 | 0,01255907 | ProcCoord24 | 0,00561976 | ProcCoord28 | 0,01109528 |
| ProcCoord21 | -0,01853612 | ProcCoord25 | 0,02072573 | ProcCoord29 | 0,05896640 |
| ProcCoord22 | 0,00701938 | ProcCoord26 | 0,00817399 | ProcCoord30 | 0,04630283 |

Sums of squares

Total SS: 0,15826903

Predicted SS: 0,00725683

Residual SS: 0,15101220

% predicted: 4,5851%

Permutation test against the null hypothesis of independence

Number of randomization rounds: 10000

P-value: 0,7252

## RESULTS OF REGRESSION ANALYSIS FOR CAUDAL PROCESSES IN VENTRAL VIEW

Total sample size: 10

Regression Coefficients

| | | | | | | | |
|---|---|---|---|---|---|---|---|
| x1 | -0,09933161 | y5 | 0,07373439 | x10 | -0,10977007 | y14 | 0,00362126 |
| y1 | 0,05764042 | x6 | -0,03838539 | y10 | -0,13473069 | x15 | 0,12672128 |
| x2 | -0,00880860 | y6 | 0,02141083 | x11 | -0,06761618 | y15 | 0,02421887 |
| y2 | 0,11728950 | x7 | -0,05202531 | y11 | -0,08519389 | x16 | 0,15812565 |
| x3 | -0,03135756 | y7 | -0,04169073 | x12 | -0,00634434 | y16 | 0,04295406 |
| y3 | 0,03322206 | x8 | -0,06920797 | y12 | -0,04789647 | x17 | 0,18719745 |
| x4 | -0,00181375 | y8 | -0,09591393 | x13 | 0,04164696 | y17 | 0,06346018 |
| y4 | 0,11558508 | x9 | -0,09432280 | y13 | -0,01909351 | | |
| x5 | -0,02307839 | y9 | -0,12861742 | x14 | 0,08837061 | | |

Sums of squares (the sums of squares below are within-group SSs)

Total SS: 0,23992373

Predicted SS: 0,13673385

Residual SS: 0,10318988

% predicted: 56,9905%

Permutation test against the null hypothesis of independence

Number of randomization rounds: 10000

P-value: 0,0002

## FIGURES

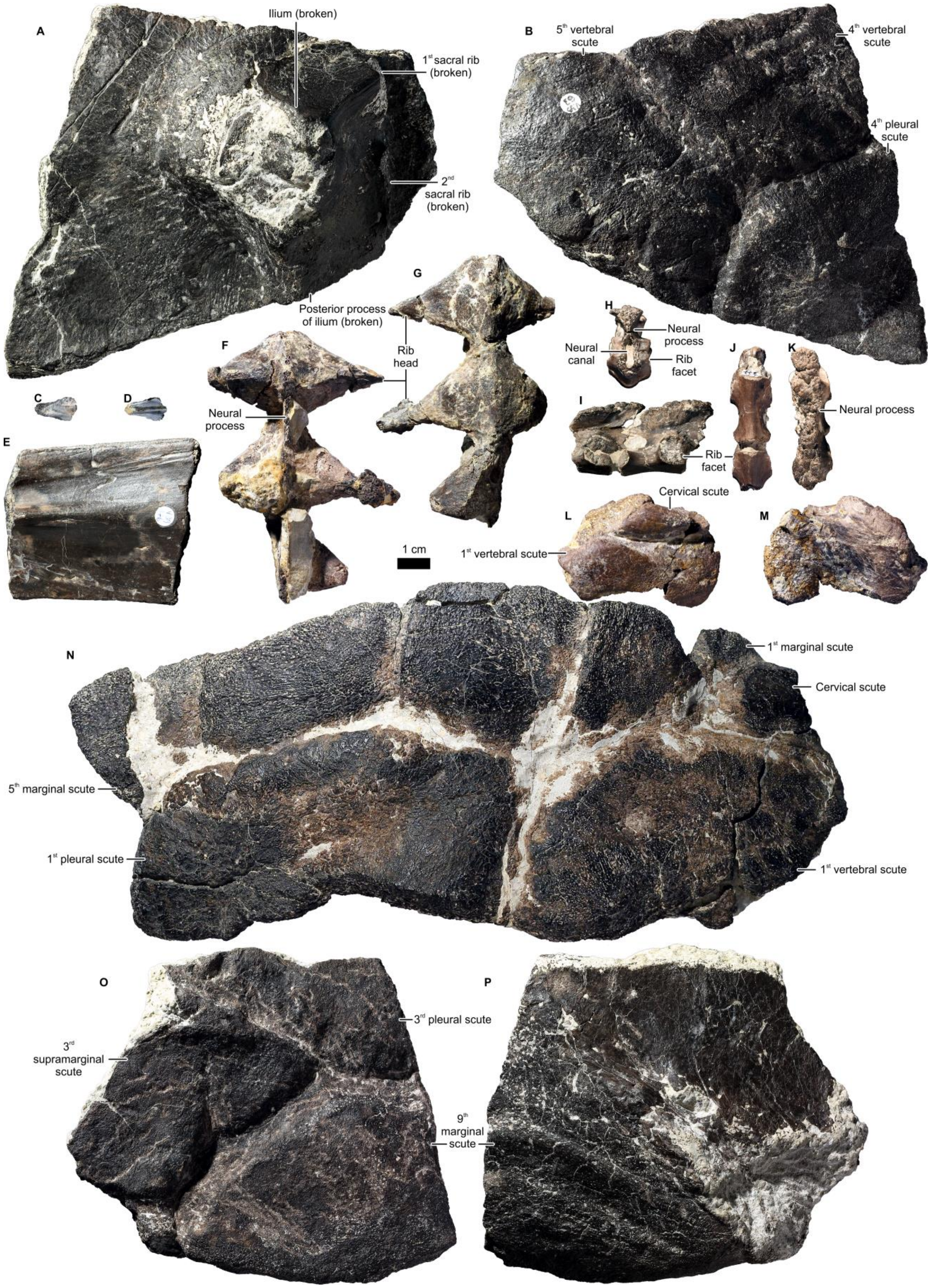


**Fig. S1.** Carapace variability of *Proterochersis porebensis*.

**A–B:** ZPAL V.39/63, carapace fragment of a large specimen with attached ilium in (**A**) visceral and (**B**) external view.

**C–D:** ZPAL V.39/381, fragmentary costal of a (?)hatchling in (**C**) external and (**D**) visceral view.

**E:** ZPAL V.39/176, costal fragment of a large specimen in visceral view.

**F–G:** ZPAL V.39/378, fragmentary dorsal vertebral column in (**F**) dorsal and (**G**) ventral view.

**H–K:** ZPAL V.39/377, fragmentary dorsal vertebral column of a juvenile in (**H**) cross-section (caudal view at a broken vertebra), (**I**) lateral right, (**J**) ventral, and (**K**) dorsal view.
**L–M:** ZPAL V.39/390, cranial part of a carapace in (**L**) external and (**M**) visceral view.
**N:** ZPAL V.39/57, cranial part of a carapace in external view.
**O–P:** ZPAL V.39/60, carapace fragment in (**O**) external and (**P**) visceral view.

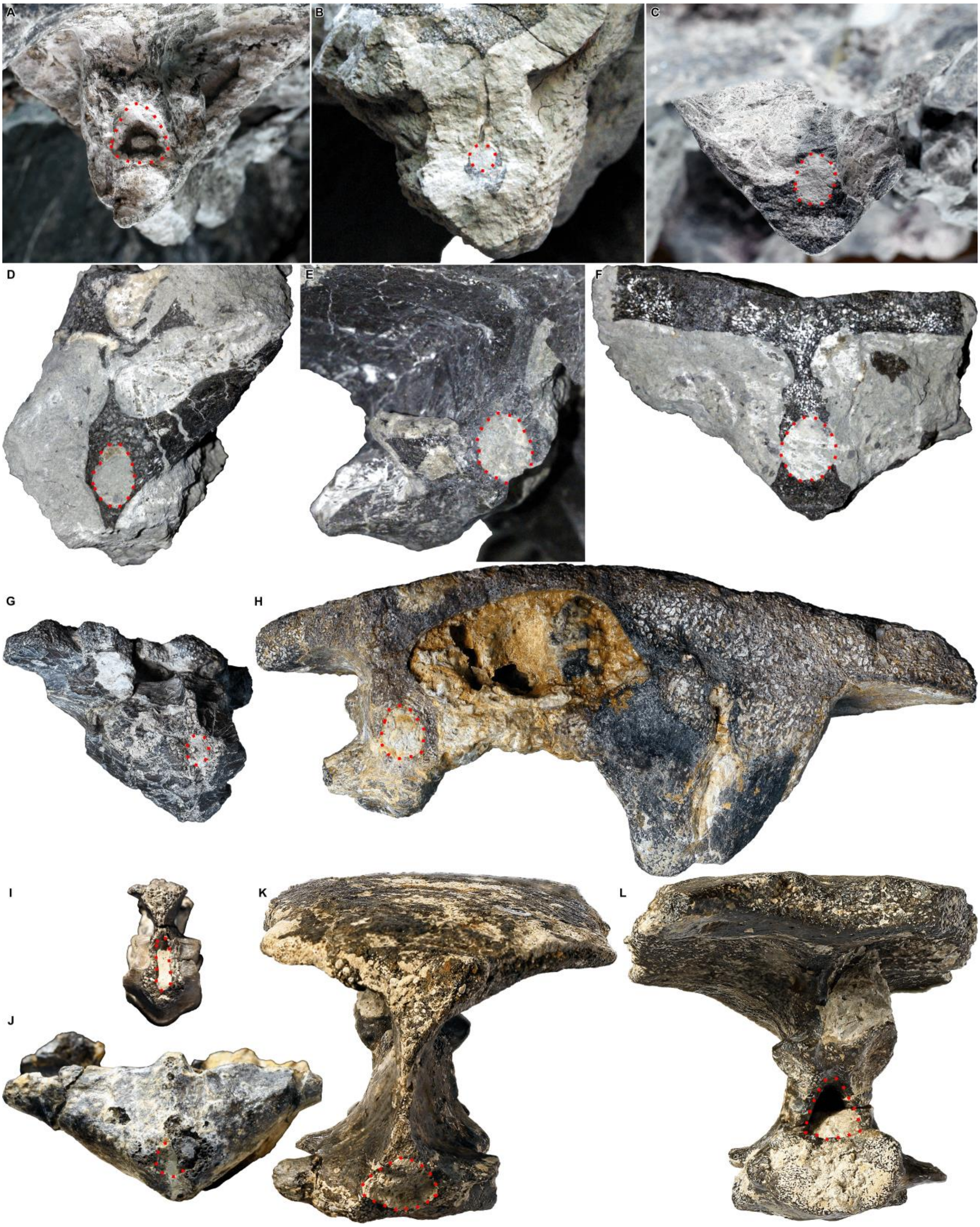


**Fig. S2.** Shape of the neural canal in *Proterochersis porebensis*.

**A–B:** ZPAL V.39/48, (**A**) cranial opening of the dorsal neural canal and (**B**) cross-section through the mid-dorsal vertebral column.

**C–F:** ZPAL V.39/49, cross-sections through the vertebral column in (**C**) cranial part of the trunk, (**D**) mid-section of the trunk, (**E**) cranial part of the sacrum, and (**F**) mid-section of the sacrum.

**G:** ZPAL V.39/169, fragmentary dorsal vertebral column in caudal view.

**H:** ZPAL V.39/370, cross-section through the first sacral vertebra in cranial view.

**I:** ZPAL V.39/377, of cross-section a dorsal vertebra in caudal view.

**J:** ZPAL V.39/378, fragmentary dorsal vertebral column in (?)cranial view.

**K–L:** ZPAL V.39/402, (**K**) damaged last dorsal vertebra in cranial view and (**P**) second sacral vertebra in caudal view.

Specimens not to scale. Outlines of the neural canals indicated by red dotted lines.

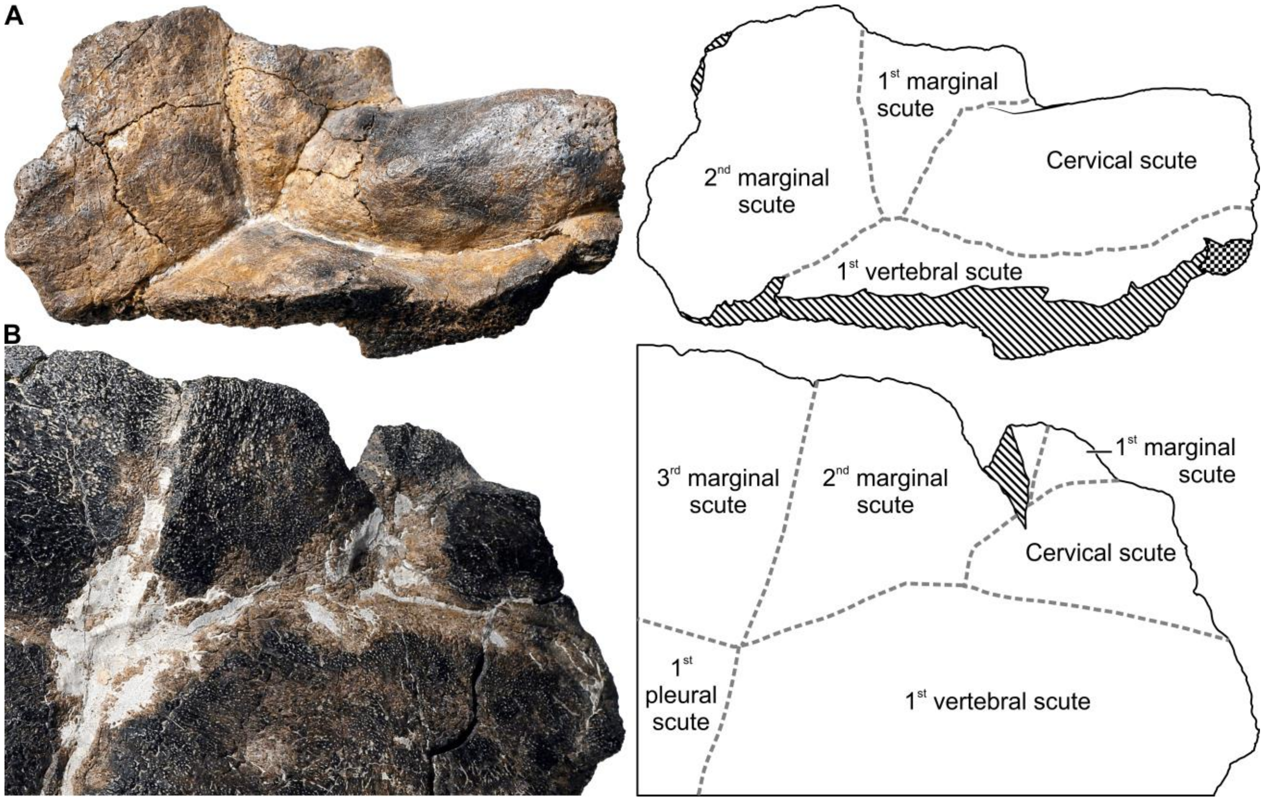


**Fig. S3.** Layout of cranial carapacial scutes in *Proterochersis porebensis*.

**A:** ZPAL V.39/22.

**B:** ZPAL V.39/57.

Specimens not to scale.

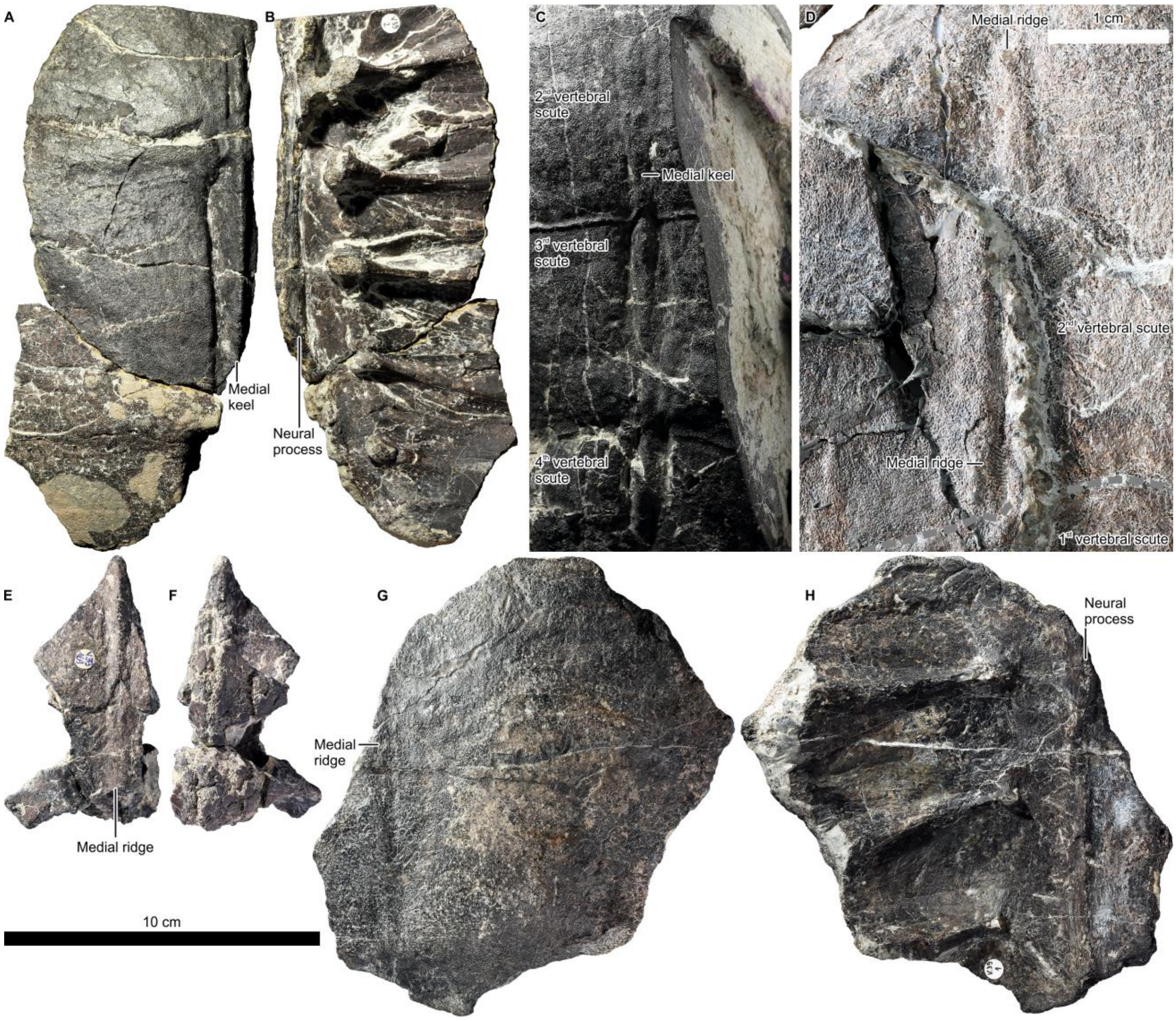


**Fig. S4.** Middorsal structures of *Proterochersis porebensis*.

**A–B:** ZPAL V.39/2, carapace fragment in (**A**) external and (**B**) visceral view.

**C:** ZPAL V.39/34, close-up on middorsal carapacial keel in dorsal view.

**D:** ZPAL V.39/72, close-up on middorsal carapacial ridge in anterodorsal view.

**E–F:** ZPAL V.39/169, carapace fragment with part of dorsal vertebral column in (**E**) dorsal and (**F**) ventral view.

**G–H:** ZPAL V.39/1, carapace fragment in (**G**) external and (**H**) visceral view.

**A–B** and **E–H** in the same scale.

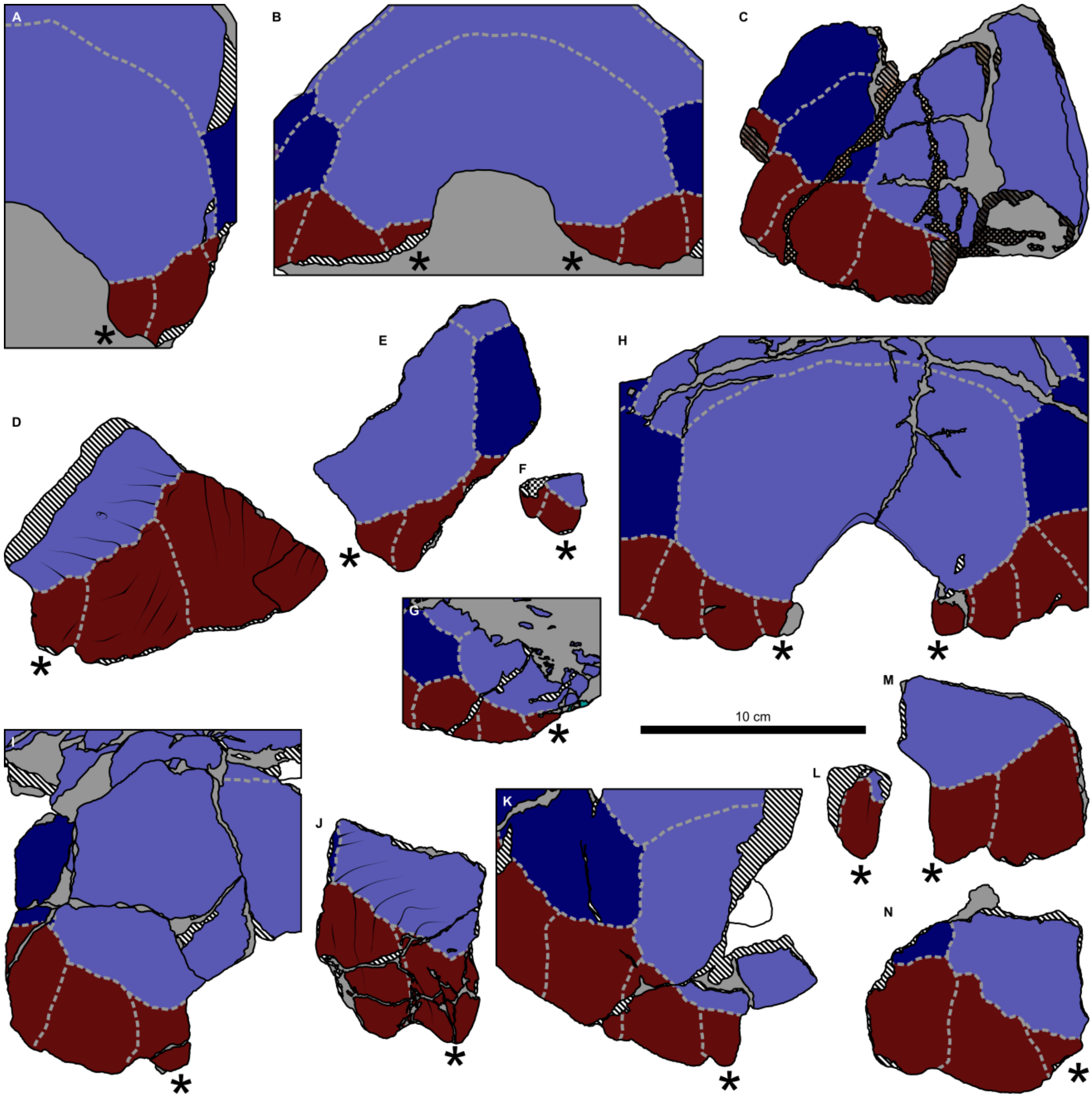


**Fig. S5.** Caudal regions of carapace of (**A–B**) *Proterochersis robusta* and (**C–N**) *P. porebensis* in external view.

**A:** CSMM uncat.
**B:** SMNS 17561.
**C:** SMNS 17755a.
**D:** ZPAL V.39/6.
**E:** ZPAL V.39/18.
**F:** ZPAL V.39/23.
**G:** ZPAL V.39/34.
**H:** ZPAL V.39/48.
**I:** ZPAL V.39/49.
**J:** ZPAL V.39/59.
**K:** ZPAL V.39/72.
**L:** ZPAL V.39/213.
**M:** ZPAL V.39/380.
**N:** ZPAL V.39/386.

Last marginals indicated by **asterisk**.

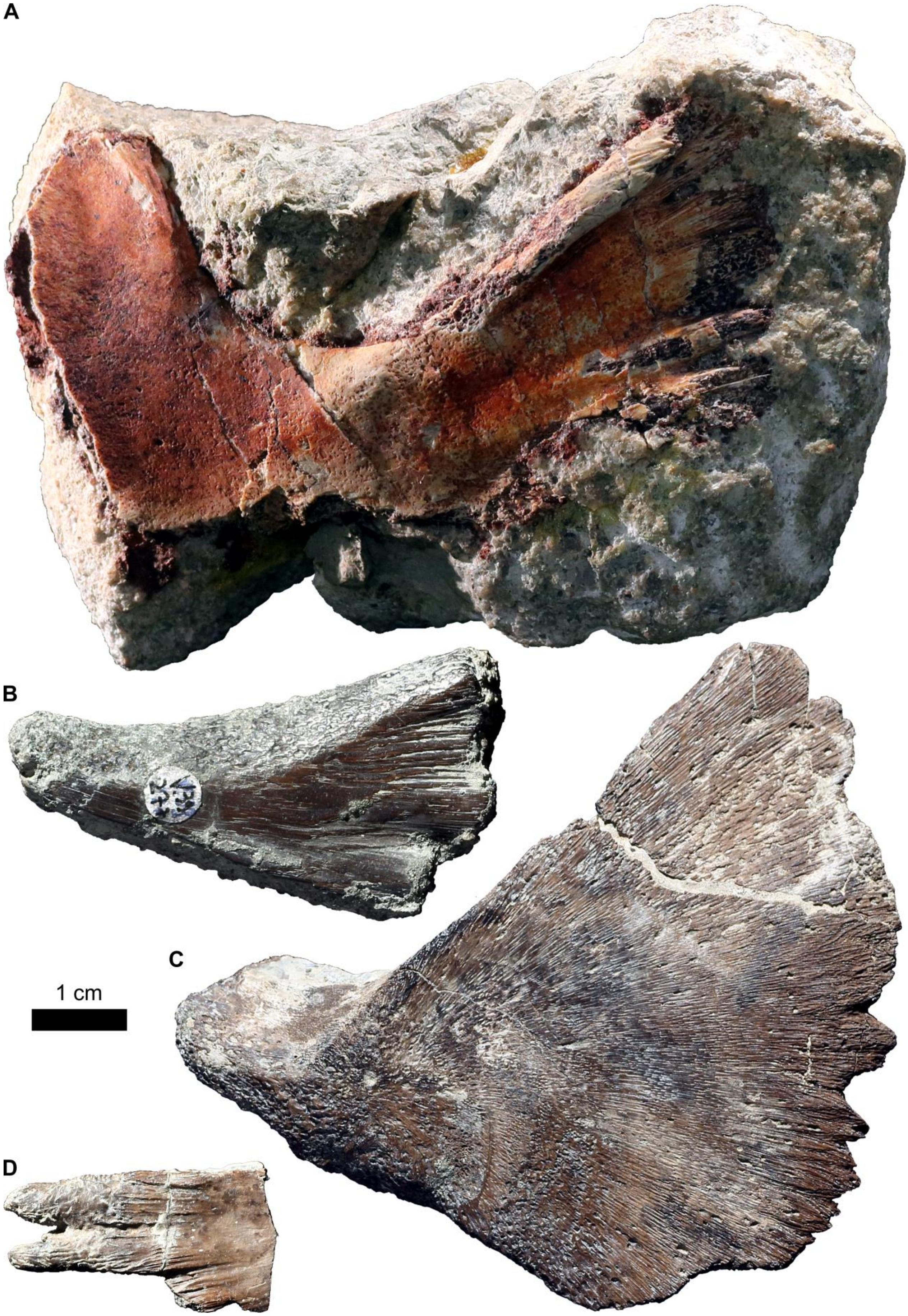


**Fig. S6.** Plastral bones of young individuals of (**A**) *Proterochersis robusta* and (**B–D**) *P. porebensis*.
**A:** SMNS 81917, hyoplastron or hypoplastron.
**B:** ZPAL V.39/277, xiphiplastron or mesoplastron.
**C:** ZPAL V.39/197, hyoplastron in external view.
**D:** ZPAL V.39/384.

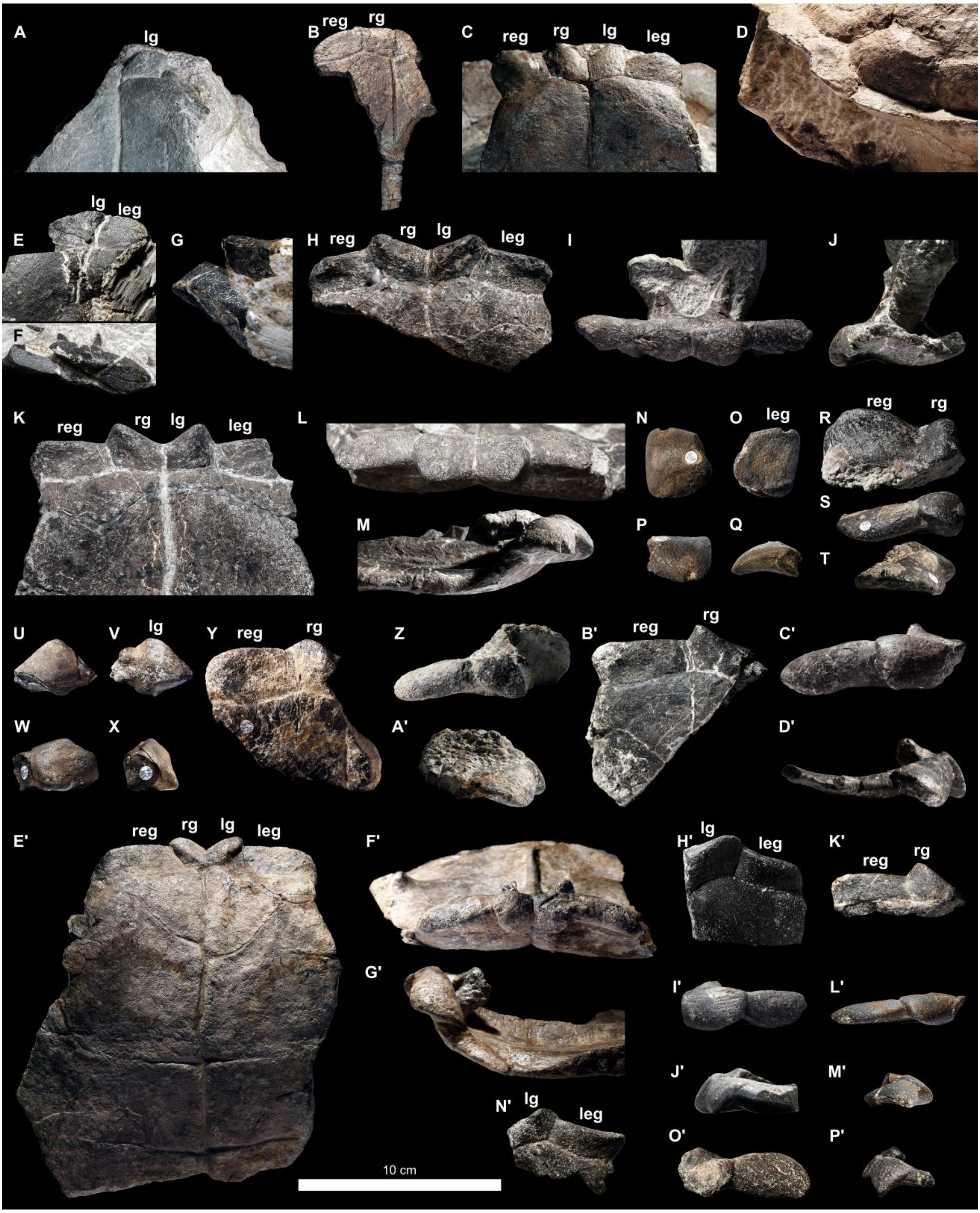


**Fig. S7.** Gular plastral regions of (**A–D**) *Proterochersis robusta* and (**E–M'**) *P. porebensis*.

**A:** CSMM uncat. in ventral view.

**B:** SMNS 16603, plastron in ventral view.

**C–D:** SMNS 17561 in (**C**) ventral and (**D**) lateral left view.

**E–G:** ZPAL V.39/34 in (**E**) ventral, (**F**) cranial, and (**G**) lateral left view.

**H–J:** ZPAL V.39/48 in (**H**) ventral, (**I**) cranial, and (**J**) lateral left view.

**K–M:** ZPAL V.39/49 in (**K**) ventral, (**L**) cranial, and (**M**) lateral right view.

**N–Q:** ZPAL V.39/186, extragular tubercle in (**N**) dorsal, (**O**) ventral, (**P**) cranial, and (**Q**) lateral left view.

**R–T:** ZPAL V.39/187 in (**R**) ventral, (**S**) cranial, and (**T**) lateral right view.

**U–X:** ZPAL V.39/189, gular tubercle in (**U**) dorsal, (**V**) ventral, (**W**) cranial, and (**X**) lateral right view.

**Y–A':** ZPAL V.39/333 in (**Y**) ventral, (**Z**) cranial, and (**A'**) lateral right view.

**B'–D':** ZPAL V.39/379 in (**B'**) ventral, (**C'**) cranial, and (**D'**) lateral right view.

**E'–G':** ZPAL V.39/385 in (**E'**) ventral, (**F'**) cranial, and (**G'**) lateral left view.

**H′–J′:** ZPAL V.39/387 in (**H′**) ventral, (**I′**) cranial, and (**J′**) lateral left view.
**K′–M′:** ZPAL V.39/388 in (**K′**) ventral, (**L′**) cranial, and (**M′**) lateral right view.
**N′–P′:** ZPAL V.39/420 in (**N′**) ventral, (**O′**) cranial, and (**P′**) lateral right view.

Abbreviations:

**leg** – left extragular scute.
**lg** – left gular scute.
**reg** – right extragular scute.
**rg** – right gular scute.

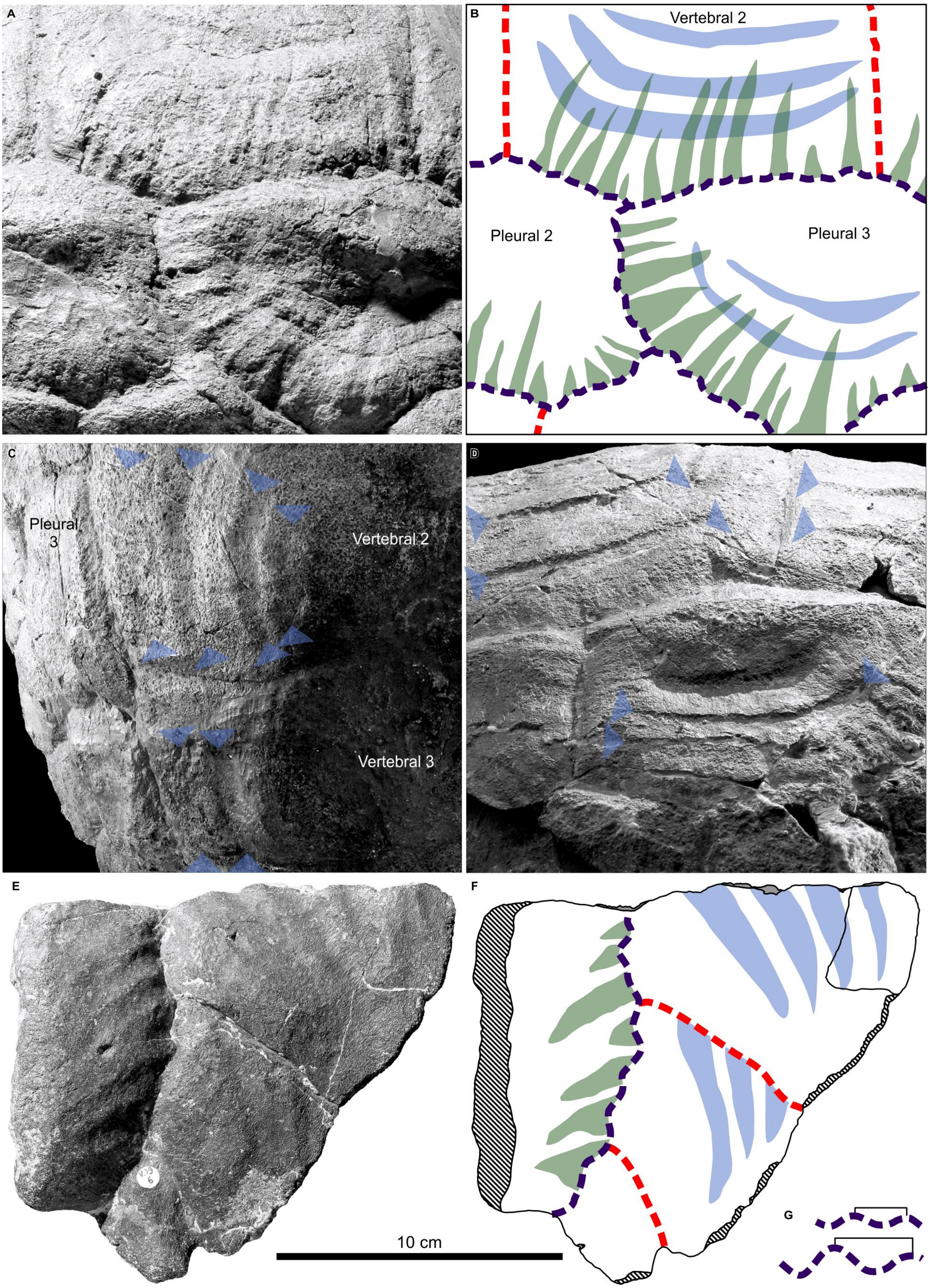


**Fig. S8.** Superficial scute features in the carapace of (**A–D**) *Proterochersis robusta* and (**E–F**) *P. porebensis*.

**A–C:** SMNS 17561 in (**A–B**) lateral left and (**C**) dorsal view.

**D:** SMNS 17930 in lateral right view.

**E–F:** ZPAL V.39/6 in external view.

**G:** Schematic representation of an undulating (sinuous) sulcus in smaller (supposedly younger, top) and larger (supposedly older specimens, bottom) individual. The frequency decreases and the amplitude of the undulations increases with the size of the specimen.

Photographs black and white for better contrast. Straight (non-undulating) sulci indicated by **red** dashed lines, undulating (sinuous) sulci indicated by **purple** dashed lines, growth marks indicated in **blue**, radial striation indicated in **green**. Note that the edges of growth marks and striations are usually ill-defined, so the layout presented here is approximate.

## REFERENCES

**Berry, J. F. & Shine, R.** 1980. Sexual size dimorphism and sexual selection in turtles (order Testudines). *Oecologia*, **44**, 185–191.

**Bookstein, F. L.** 1997. *Morphometric tools for landmark data: Geometry and biology*. Cambridge University Press.

**Brophy, T. R.** 2006. Allometry and sexual dimorphism in the snail-eating turtle *Malayemys macrocephala* from the Chao Phraya River Basin of central Thailand. *Chelonian Conservation and Biology*, **5**, 159–165, doi: 10.2744/1071-8443(2006)5[159:AASDIT]2.0.CO;2.

**Cadena, E. A., Jaramillo, C. A. & Bloch, J. I.** 2013. New material of the platychelyid turtle *Notoemys zapatocaensis* from the Early Cretaceous of Colombia; Implications for understanding Pleurodira evolution. *In*: Brinkman, D. B., Holroyd, P. A. & Gardner, J. D. (eds) *Morphology and Evolution of Turtles*. Springer Science+Business Media, Dordrecht, 105–120.

**Ceballos, C. P. & Iverson, J. B.** 2014. Patterns of sexual size dimorphism in Chelonia: Revisiting Kinosternidae. *Biological Journal of the Linnean Society*, **111**, 806–809, doi: DOI 10.1111/j.1095-8312.2012.02015.x.

**Chiari, Y. & Claude, J.** 2011. Study of the carapace shape and growth in two Galápagos tortoise lineages. *Journal of Morphology*, **272**, 379–386, doi: 10.1002/jmor.10923.

**Cignoni, P., Callieri, M., Corsini, M., Dellepiane, M., Ganovelli, F. & Ranzuglia, G.** 2008. MeshLab: An open-source mesh processing tool. In: *Sixth Eurographics Italian Chapter Conference*. 129–136, doi: 10.2312/LocalChapterEvents/ItalChap/ItalianChapConf2008/129-136.

**Cordero, G. A.** 2018. Is the pelvis sexually dimorphic in turtles? *The Anatomical Record*, 1–22, doi: 10.1002/ar.23831.

**de Broin, F.** 1984. *Proganochelys ruchae* n.sp., chélonien du Trias supérieur de Thaïlande. *Studia Palaeocheloniologica*, **1**, 87–97.

**Fraas, E.** 1913. *Proterochersis*, eine pleurodire Schildkröte aus dem Keuper. *Jahreshefte des Vereins für Vaterlandische Naturkunde in Württemberg*, **69**, 13–30.

**Gaffney, E. S.** 1986. Triassic and Early Jurassic turtles. *In*: Padian, K. (ed.) *The Beginning of the Age of Dinosaurus. Faunal Change across the Triassic-Jurassic Boundary*. Press Syndicate of the University of Cambridge, Cambridge, 183–187.

**Gaffney, E. S.** 1990. The comparative osteology of the Triassic turtle *Proganochelys*. *Bulletin of the American Museum of Natural History*, **194**, 1–263.

**Karl, H.-V. & Tichy, G.** 2000. *Murrhardtia staeschei* n. gen. n. sp. – eine neue Schildkröte aus der Oberen Trias von Süddeutschland. *Joannea Geologie und Paläontologie*, **2**, 57–72.

**Keswick, T. & Hofmeyr, M. D.** 2015. Sexual dimorphism and geographic variation in the morphology of a small southern African tortoise *Psammobates oculifer*. *Amphibia-Reptilia*, **36**, 55–64, doi: 10.1163/15685381-00002976.

**Klingenberg, C. P.** 2009. Morphometric integration and modularity in conFigurations of landmarks: Tools for evaluating a priori hypotheses. *Evolution and Development*, **11**, 405–421, doi: 10.1111/j.1525-142X.2009.00347.x.

**Klingenberg, C. P.** 2011. MorphoJ: An integrated software package for geometric morphometrics. *Molecular Ecology Resources*, **11**, 353–357, doi: 10.1111/j.1755-0998.2010.02924.x.

**Leuteritz, T. E. J. & Gantz, D. T.** 2013. Sexual Dimorphism in Radiated Tortoises (*Astrochelys radiata*). *Chelonian Research Monographs*, **6**, 105–112, doi: 10.3854/crm.6.a18p105.

**Lichtig, A. J. & Lucas, S.** 2017. Sutures of the shell of the Late Cretaceous-Paleocene baenid turtle *Denazinemys*. *Neues Jahrbuch für Geologie und Paläontologie - Abhandlungen*, **283**, 1–8, doi: 10.1127/njgpa/2017/0622.

**Młynarski, M.** 1976. *Encyclopedia of Paleoherpetology. Part 7. Testudines*. Gustav Fischer Verlag, Stuttgart and New York, 130 pp.

**Pritchard, P. C. H.** 2008. Evolution and structure of the turtle shell. *In*: Wyneken, J., Godfrey, M. H. & Bels, V. (eds)

*Biology of Turtles*. CRC Press, Boca Raton, London & New York, 46–83.

**Rohlf, F. J.** 2015. The tps series of software. *Hystrix*, **26**, 1–4, doi: 10.4404/hystrix-26.1-11264.

**Scheyer, T. M. & Sander, P. M.** 2007. Shell bone histology indicates terrestrial palaeoecology of basal turtles. *Proceedings of the Royal Society of London B: Biological Sciences*, **247**, 1885–1893.

**Stromer, E. F.** 1912. *Lehrbuch Der Paläozoologie. II. Teil: Wirbeltiere*. B.G. Teubner, Leipzig and Berlin, 325 pp.

**Sulej, T., Niedźwiedzki, G. & Bronowicz, R.** 2012. A new Late Triassic vertebrate fauna from Poland with turtles, aetosaurs, and coelophysoid dinosaurs. *Journal of Vertebrate Paleontology*, **32**, 1033–1041, doi: 10.1080/02724634.2012.694384.

**Sullivan, P. M. & Joyce, W. G.** 2017. The shell and pelvic anatomy of the Late Jurassic turtle *Platychelys oberndorferi* based on material from Solothurn, Switzerland. *Swiss Journal of Palaeontology*, **136**, 323–343.

**Szczygielski, T.** 2017. Homeotic shift at the dawn of the turtle evolution. *Royal Society Open Science*, **4**, 160933, doi: http://dx.doi.org/10.1098/rsos.160933.

**Szczygielski, T. & Sulej, T.** 2016. Revision of the Triassic European turtles *Proterochersis* and *Murrhardtia* (Reptilia, Testudinata, Proterochersidae), with the description of new taxa from Poland and Germany. *Zoological Journal of the Linnean Society*, **177**, 395–427, doi: 10.1111/zoj.12374.

**Szczygielski T. & Sulej T.** In press. The early composition and evolution of the turtle shell (Reptilia, Testudinata). *Palaeontology*.

**White, J. B. & Murphy, G. G.** 1973. The reproductive cycle and sexual dimorphism of the common snapping turtle, *Chelydra serpentina serpentlna*. *Herpetologica*, **29**, 240–246.

**Wibbels, T., Owens, D. W. & Rostal, D. C.** 1991. Soft plastra of adult male sea turtles: An apparent secondary sexual characteristic. *Herpetological Review*, **22**, 47–49.

**Wild, R.** 1987. Die Tierwelt der Keuperzeit (unter besonderer Berücksichtigung der Wirbeltiere. *Natur an Rems und Murr*, **6**, 17–43.

**Wu, C.** 2011. VisualSFM: A visual structure from motion system. Available at http://ccwu.me/vsfm/ (acessed 22 June 2018).

**Wyneken, J.** 2001. *The Anatomy of Sea Turtles*. U.S. Department of Commerce NOAA Technical Memorandum NMFS-SEFSC-470, 172 pp.
