# Supplementary material for: Shell variability in the stem turtles Proterochersis spp
Source: PeerJ. 2018 Dec 21;6:e6134. doi: 10.7717/peerj.6134 (PMC6305121; doi:10.7717/peerj.6134)
Supplement: Supplemental Information 2 — The numbers of specimens are from top to bottom ZPAL V.39/34, ZPAL V.39/48, ZPAL V.39/49, ZPAL V.39/187, ZPAL V.39/333, ZPAL V.39/379, ZPAL V.39/385, ZPAL V.39/387, ZPAL V.39/388, and ZPAL V.39/420. [file peerj-06-6134-s002.pdf]

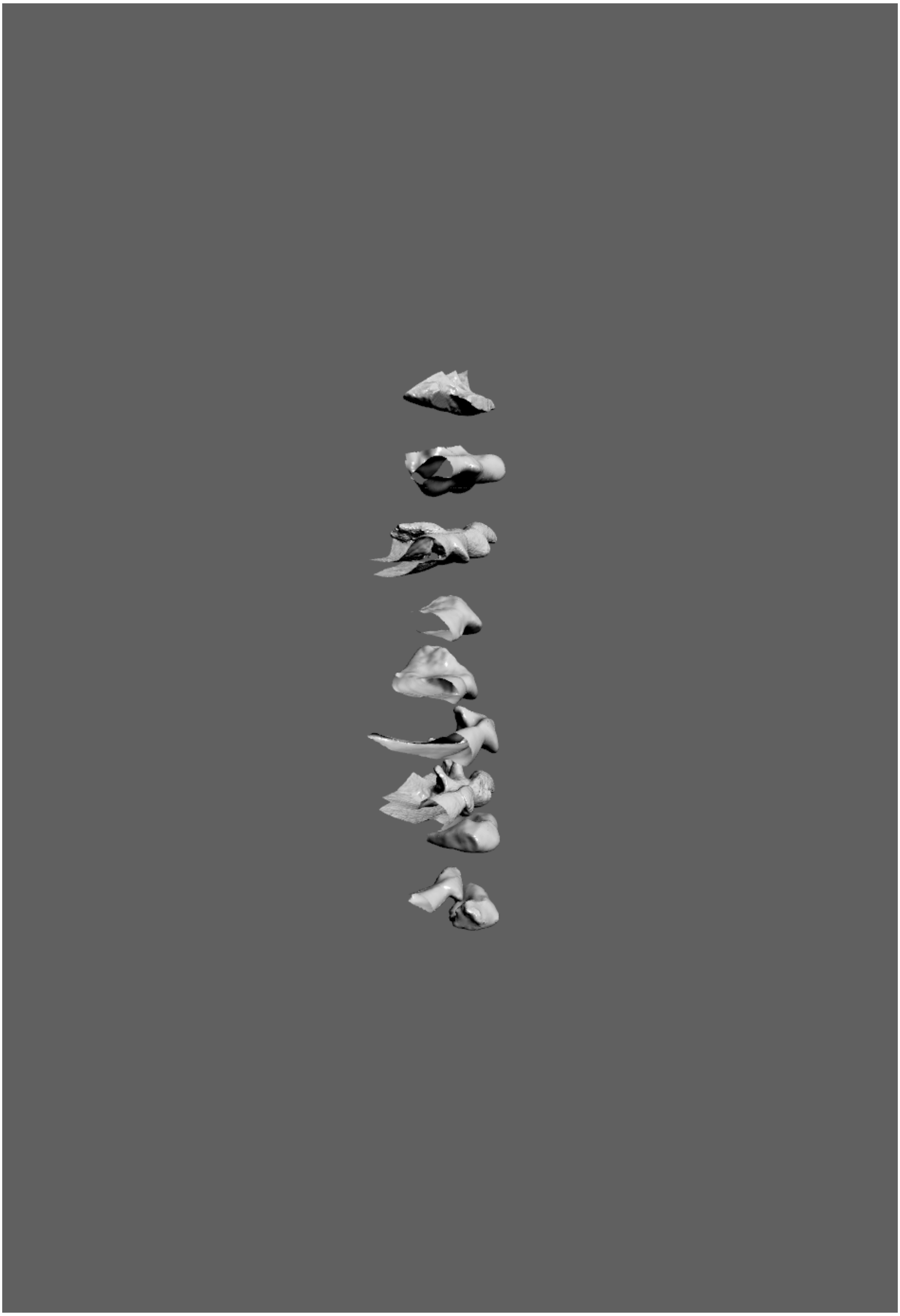

Article S2. Models of anterior regions of the turtle shells of *Proterochersis porebensis*, with sectioned extragulars.
